# Stochastic variation of transcript abundance in C57BL/6J mice

**DOI:** 10.1186/1471-2164-12-167

**Published:** 2011-03-30

**Authors:** Peter T Vedell, Karen L Svenson, Gary A Churchill

**Affiliations:** 1 The Jackson Laboratory, 600 Main Street, Bar Harbor, Maine 04609, USA

## Abstract

**Background:**

Transcripts can exhibit significant variation in tissue samples from inbred laboratory mice. We have designed and carried out a microarray experiment to examine transcript variation across samples from adipose, heart, kidney, and liver tissues of C57BL/6J mice and to partition variation into within-mouse and between-mouse components. Within-mouse variance captures variation due to heterogeneity of gene expression within tissues, RNA-extraction, and array processing. Between-mouse variance reflects differences in transcript abundance between genetically identical mice.

**Results:**

The nature and extent of transcript variation differs across tissues. Adipose has the largest total variance and the largest within-mouse variance. Liver has the smallest total variance, but it has the most between-mouse variance. Genes with high variability can be classified into groups with correlated patterns of expression that are enriched for specific biological functions. Variation between mice is associated with circadian rhythm, growth hormone signaling, immune response, androgen regulation, lipid metabolism, and the extracellular matrix. Genes showing correlated patterns of within-mouse variation are also associated with biological functions that largely reflect heterogeneity of cell types within tissues.

**Conclusions:**

Genetically identical mice can experience different individual outcomes for medically important traits. Variation in gene expression observed between genetically identical mice can identify functional classes of genes that are likely to vary in the absence of experimental perturbations, can inform experimental design decisions, and provides a baseline for the interpretation of gene expression data in interventional studies. The extent of transcript variation among genetically identical mice underscores the importance of stochastic and micro-environmental factors and their phenotypic consequences.

## Background

Variation in transcript abundance between individuals has important implications for microarray experimental design and significance testing [1]. Ideally, microarray experiments are designed with samples from multiple individuals in each treatment group. This biological replication provides the variance estimator that is required to establish the statistical significance of between-group differences. In this study, we collected multiple samples of tissues within each of several genetically identical mice. Multiple sampling within individuals is not necessary in an experiment aimed at making between-group comparisons, but it is essential if the aim is to identify significant variation between individuals within the same experimental treatment group. An important procedural detail in this type of study is to determine how to collect and at what stage to divide the tissues to create multiple samples. In this study, we elected to split tissues immediately after dissection and before RNA extraction in order to restrict the possible sources of between-mouse variation to events that occur prior to dissection. With this experimental design, transcript variation can be decomposed into within-mouse and between-mouse variance components. Between-mouse variance reflects differences in whole-tissue transcript abundance between genetically identical mice. Within-mouse variance captures variation due to RNA extraction, array processing, and heterogeneity of gene expression within tissues, which may be amplified by dissection and tissue collection procedures.

Individual variation in gene expression can have important phenotypic consequences. However, only a few studies have previously attempted to characterize gene expression variation in genetically identical mice. Koza et al. (2006) [2] described gene expression signatures in adipose tissue that are predictive of future adiposity among genetically identical C57BL/6J mice. The use of multiple biopsy samples in this time-course study was essential to establish the link between gene expression variation and late-life adiposity. However, biopsy sampling may be subject to unexpected variation introduced by tissue heterogeneity, as we illustrate below.

Two previous studies have used multiple sampling within individuals to provide a statistical basis for detecting transcript variation between genetically identical mice. Pritchard et al. (2001) [3] examined 3 tissues in each of 6 C57BL/6J mice and reported that immune function, stress response, and hormone regulation were important sources of biological variation. Pritchard et al. (2006) [4] examined liver tissue in 3 animals from each of 5 inbred mouse strains and found that genes differentially expressed within strains were enriched for cell growth, cytokine activity, amine metabolism, and ubiquitination. In these experiments, technical replicates were obtained by splitting samples after RNA extraction. This approach confounds variation due to dissection and RNA preparation with variation between mice.

**Figure 1:**
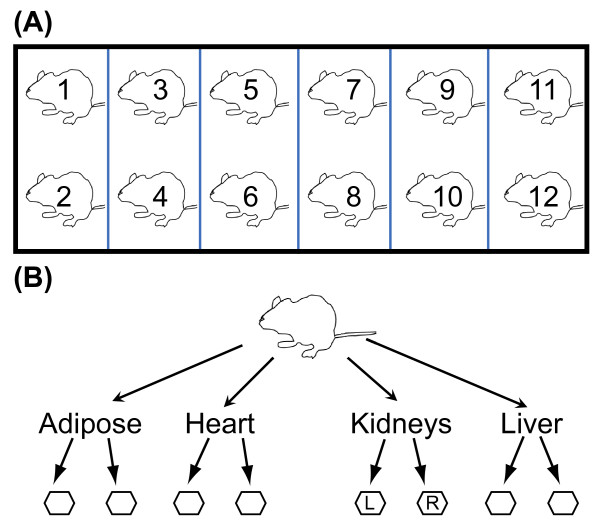
**Experimental design**. Twelve C57BL/6J male mice were co-housed as pairs in six cages from weaning until 10 weeks of age (A). Liver, heart, kidney, and adipose tissues were collected from each mouse and split into two samples per tissue per individual and processed separately to generate RNA (B). In the case of kidney, the samples consist of the entire left or right kidney, respectively. Other tissues were chopped into small pieces, which were separated before placing them into collection tubes.

We designed and carried out an experiment to study transcript abundance variation in four tissues among young adult male C57BL/6J mice (Figure 1). Our sampling design enabled us to partition the variance for each gene into within-mouse and between-mouse components, with a division line that corresponds to the step of splitting tissues. We examined within-mouse and between-mouse variation in more than 22,000 protein coding genes and identified groups of genes with shared patterns of variation that are enriched for known biological functions. To facilitate exploration of our data, we have created an on-line resource that includes graphical displays, test statistics, and gene groupings for all transcripts characterized in this study http://cgd.jax.org/individualvariation.shtml.

## Results

We performed a microarray experiment using the Illumina Sentrix^® ^Mouse-6 v1.1 BeadChip microarray platform to study transcript variation in 10-week old male C57BL/6J mice (Figure 1). Six pairs of siblings were co-housed from weaning under uniform environmental conditions. From each mouse we obtained duplicate samples of adipose (inguinal fat pad), heart, kidney, and liver tissues by splitting whole organs or tissues prior to homogenization and RNA extraction. Adipose, heart, and liver tissues were coarsely cut into pieces and divided into two samples that were homogenized separately in order to extract RNA. The left and right kidneys were also homogenized separately. We computed a decomposition of variance for each probe on the array (Methods). The within-mouse variance component captures biological variance between two dissected tissue samples as well as technical variance due to sample and microarray processing. The between-mouse variance component reflects differences between individual mice. We repeated gene expression assays on the liver samples, using the Affymetrix Whole-Transcript Mouse Gene 1.0 ST array, to provide validation on a different measurement platform.

### Expressed genes and variable genes

We declared a gene to be expressed if the probe intensity was greater than the 95^th ^percentile of the negative control probes for both samples in at least 1 of the 12 mice. A total of 12657 genes, representing 55% of the annotated probes on the array, were expressed in at least one of the four tissues. Across tissues, the number of expressed genes ranged from 8919 (39%) in liver to 11204 (49%) in adipose tissue (Table 1A).

**Table 1:** Variability of transcript abundance

		Adipose	Heart	Kidney	Liver	Total
**(A)**	**Expressed Genes**	11204(49%)	10069(44%)	10116(44%)	8919(39%)	12657(55%)
**(B)**	**Variable Genes**					
	***α *= 0.05**	3923(17%)	2654(12%)	2273(10%)	2125(9%)	6932(30%)
	***α *= 0.01**	3299(14%)	2117(9%)	1547(7%)	1539(7%)	5800(25%)
	***α* = 1e-4**	2352(10%)	1250(5%)	847(4%)	813(4%)	3939(17%)
**(C)**	**Between-Mouse Variation**					
	**FWER (p < 0.05)**	0(0%)	0(0%)	12(0%)	40(0%)	46(0%)
	**FDR (p < 0.10)**	0(0%)	0(0%)	369(2%)	2520(11%)	2674(12%)
	**1 - π_0_**	0%	0%	1%	18%	
**(D)**	**Maximal Fold Change**					
	**> 1.5**	2103(9%)	509(2%)	320(1%)	538(2%)	2690(12%)
	**> 2.0**	600(3%)	142(1%)	91(0%)	809(4%)	809(4%)
	**> 3.0**	218(1%)	50(0%)	28(0%)	36(0%)	292(1%)

Shown in this table are: The number of expressed genes out of the 22869 annotated genes on the array (A). The number of variable genes based on the 0.05, 0.01 and 0.0001 tails of the scaled *χ_2_*(23) distribution (B). The number of genes with significant between-mouse variance, based on the *F_s _*test with family-wise adjusted error (p < 0.05, Sidak step-down method [5]); false discovery rate (p < 0.10, Benjamini-Hochberg method [6]); and the estimated proportion of differentially expressed genes (1 - π_0_, using the q-value method [7]) (C). The number of genes with large maximal fold-change between mice (D). All numbers are given as percentages of the annotated genes in parentheses.

**Figure 2:**
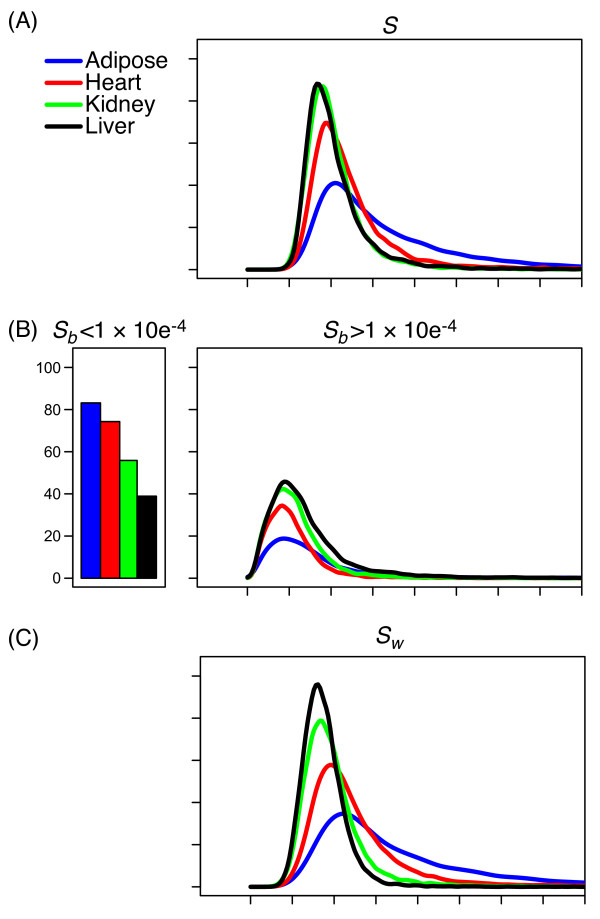
**Distribution of within- and between-mouse variance components**. The total variance (A) and estimated between-mouse (B) and within-mouse (C) variance components are shown as smoothed density histograms after square root transformation. The vertical bars (left panel of B) represent the percentage of probes with estimated variance components less than 1×10^-4^. These probes are not included in the density plot (right panel of B). Tissues are indicated by colour (Adipose: blue; Heart: red; Kidney: green; Liver: black).

We computed the total variance, *s^2^*, across all samples for each gene in each tissue (Figure 2A). Liver and kidney have relatively few genes of high variability but heart and adipose have many. We tested the hypothesis that the distribution of total variance occurred by chance using a *χ^2 ^*test (Methods) and found significantly greater variance than expected in each tissue (Table 1B). We applied coexpression network analysis to the top 2500 genes in each tissue, which we refer to as the *variable genes*.

We decomposed total variance for each gene into within-mouse (*s_w_^2^*) and between-mouse (*s_b_^2^*) components. The distribution of between-mouse variance components was similar across all four tissues (Figure 2B). Adipose tissue showed the greatest number of genes with a large within-mouse component followed by heart, kidney, and liver (Figure 2C). The variable genes include the 313 (adipose), 189 (heart), 405 (kidney), and 990 (liver) genes with the largest between-mouse variance. They also include the 1526 (adipose), 1347 (heart), 593 (kidney), and 221 (liver) genes with the largest within-mouse variance.

### Significance of between-mouse variance

Within each tissue, for each gene, we computed a test statistic to assess the significance of the between-mouse variance component relative to the within-mouse variance component. We applied a family-wise error rate correction [5] (as in Pritchard et al. (2001) [3]) and found few genes with significant between-mouse variation (Table 1C). We applied a false discovery rate (FDR) adjustment [6] (as in Pritchard et al. (2006) [4]) and found no differentially expressed genes in adipose or heart and only modest numbers in kidney (2%) and liver (11%) (Table 1C). We estimated the proportions of differentially expressed genes (1 - π_*0*_) using the q-value software [7] and found similar results (Table 1C; [Additional files 1, 2: Supplemental Figure S1]).

A different picture of the variability in gene expression across tissues emerges when we look at the maximal fold change between mice (Table 1D). In adipose, 2.6% of all genes exhibit maximal fold changes greater than 2, whereas 0.4-0.6% of all genes show fold changes this large in the other three tissues. Although the fold-change is not a statistical criterion, the differences across tissues are dramatic. There are many genes with large maximal fold changes between mice but, particularly in adipose tissue, these same genes also have large within-mouse variance, which reduces their statistical significance.

### Variable genes form clusters that are enriched for specific biological functions

**Figure 3:**
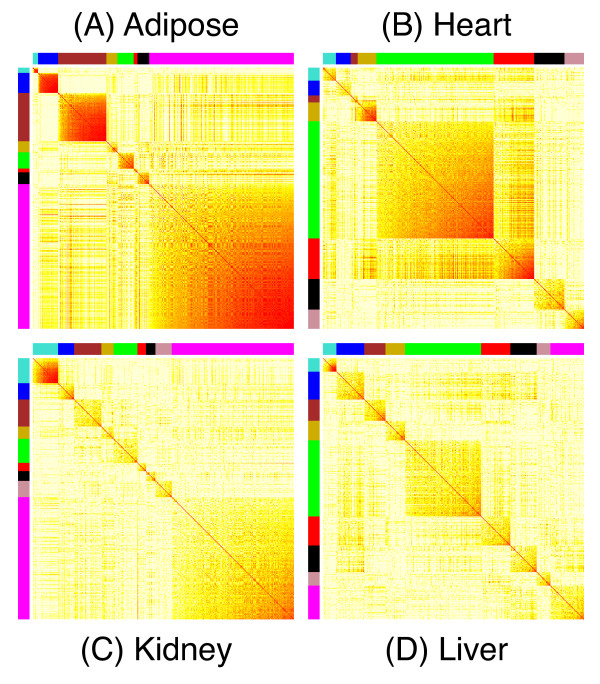
**Co-expression module clustering of variable genes**. The topographical overlap similarity [9] of gene pairs is shown as a heatmap for adipose (A), heart (B), kidney (C), and liver (D). Gene pairs with low similarity are shown in yellow, and those with high similarity are shown in red. Coexpression modules define highly connected sets of correlated expression profiles as indicated by colour codes in the margins of the maps. The colour names are given in Table 2. Genes are ordered within module from left to right by increasing topographical overlap with the module eigengene. Genes that are not assigned to modules are not included in this plot.

**Figure 4:**
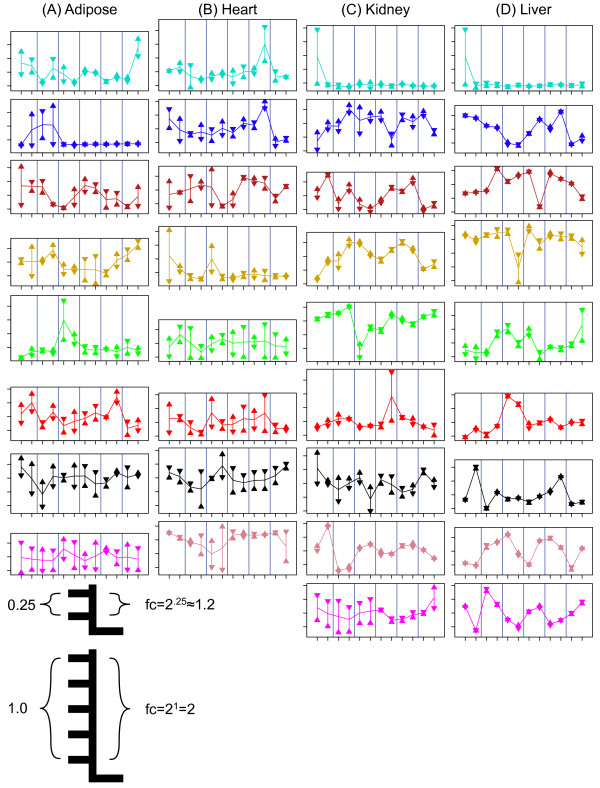
**Transcript abundance profiles of co-expression modules**. Module eigengenes representing average transcript abundance across genes are shown for the modules listed in Table 2. Mice are indexed from 1 to 12 and vertical partitions indicate cage pairings. Eigengene profiles are zero-centered and represent an average log_2 _fold-change (fc) across all genes in the module. All panels are shown with the same y-axis scaling for ease of comparison. Horizontal lines connect mean values for each mouse. Vertical line segments connect the two within-mouse samples. Sample 1 is indicated by the upright triangle and sample 2 by the inverted triangle. For adipose, heart, and liver, samples should not be compared across mice or across tissues, as there is no correspondence between sample labels at this level of the experiment. In the kidney, sample 1 is the left kidney and sample 2 is the right kidney. Modules with 25 or fewer genes are not shown.

We used co-expression network analysis [8,9] to cluster the variable genes into modules with correlated patterns of expression (Methods) (Figure 3). Module sizes ranged from 34 to 1340 genes with an average module size of 215 genes (Table 2). We identified 8 to 9 modules in each tissue comprising 97% (adipose), 80% (heart), 61% (kidney), and 54% (liver) of the variable genes. For each module, we applied principal components analysis to compute a module eigengene [10] that represents the dominant pattern of variation (Figure 4). The percentage of variation explained by the module eigengene ranges from 47% to 88%, indicating that the eigengenes are representative of expression profiles of the individual genes in each module. In the following, we refer to modules using a colour code within each tissue (Table 2).

**Table 2:** Module highlights

	Module	Data-base	Functional Category	In Module	In Category	Overlap	%	p-value	*c*
Adipose	turquoise	GO_MF	endopeptidase inhibitor activity	52	55	8	15	7E-25	0.44
	blue	GO_BP	epidermis development	184	40	12	30	3E-23	0.00
	brown	GO_CC	contractile fiber	450	56	39	70	2E-74	0.07
	gold	GO_MF	acyl group transferase activity	102	64	10	16	6E-19	0.12
	green	GO_MF	cytokine activity	147	53	15	28	6E-32	0.28
	red	GO_BP	Apoptosis	34	245	6	2	7E-06	0.42
	black	GO_BP	energy pathways	110	104	10	10	2E-09	0.00
	magenta	GO_BP	immune system process	1340	324	158	49	1E-34	0.00

Heart	turquoise	GO_BP	inflammatory response	102	103	14	14	6E-21	0.17
	blue	GO_BP	anti-apoptosis	115	29	5	17	3E-10	0.20
	brown	GO_CC	antigen presentation, exogenous antigen	52	15	9	60	1E-75	0.03
	gold	KEGG	leukocyte activation	145	116	18	55	1E-25	0.00
	green	GO_CC	extracellular matrix	898	111	57	51	2E-26	0.00
	red	GO_BP	monosaccharide biosynthetic process	313	18	8	44	1E-13	0.00
	black	GO_CC	ubiquitin ligase complex	237	14	5	36	1E-10	0.00
	pink	GO_CC	mitochondrial membrane part	147	27	12	44	1E-55	0.00

Kidney	turquoise	GO_BP	acute inflammatory response	146	46	17	37	1E-47	0.01
	blue	GO_CC	extracellular matrix	94	111	22	20	8E-51	0.42
	brown	GO_MF	carboxylic acid transmembrane transport	159	19	4	21	3E-07	0.58
	gold	GO_BP	antigen presentation, exogenous antigen	72	15	7	47	2E-56	0.57
	green	GO_CC	mitochondrial outer membrane	141	34	5	15	1E-06	0.59
	red	GO_MF	nucleoside-triphosphatase activity	46	159	4	3	4E-03	0.13
	black	GO_BP	fatty acid biosynthetic process	59	42	5	12	3E-12	0.10
	pink	GO_BP	down regulation of signal transduction	92	50	6	12	6E-12	0.69
	magenta	KEGG	oxidative phosphorylation	711	50	19	38	7E-08	0.00

Liver	turquoise	GO_CC	Myofibril	70	54	9	17	2E-26	0.02
	blue	GO_MF	enzyme regulator activity	146	227	13	6	1E-03	0.77
	brown	GO_CC	extracellular matrix	112	111	13	12	2E-13	0.74
	gold	GO_BP	cholesterol biosynthetic process	101	18	6	33	5E-26	0.44
	green	GO_MF	selenium binding	395	14	5	36	7E-06	0.39
	red	GO_BP	cytokine-mediated signalling pathway	152	21	5	24	5E-10	0.72
	black	GO_BP	blood vessel development	138	109	9	8	2E-05	0.78
	pink	GO_BP	antigen presentation, exogenous antigen	69	15	6	40	4E-45	0.79
	magenta	GO_BP	ribosome biogenesis	176	22	5	23	1E-08	0.74

Best enrichment scores of co-expression modules are shown. Within each tissue, modules are listed by the colour code orders of Figure 3. The number of genes in the module, the number of variable genes in the highest scoring functional category, the overlap between these two sets of genes, the enrichment p-value for the highest scoring category [11], and the median intraclass correlation, *c*, for genes in the module are shown. Only categories with between 10 and 500 genes and a minimum overlap of 4 genes were considered.

For each gene, we computed the intraclass correlation coefficient, *c *≡ *s_b_^2^/*(*s_w_^2 ^+ s_b_^2^*), which is the proportion of total variance attributable to the between-mouse component. Median values by module ranged from *c *= 0.00 (8 modules) to *c *= 0.79 (liver-pink) (Table 2). Kidney and liver, respectively, have 5 and 8 modules with high intraclass correlation (*c *≥ 0.35) indicating substantial between-mouse variance while adipose has two and heart has no modules with high intraclass correlation (*c *≥ 0.35). Each tissue also has at least one module with low intraclass correlation (*c ≤ *0.02) indicating that differences between samples within mice are greater than differences between mice.

For each module, we computed enrichment scores [11] for the GO biological process, cellular component, and molecular function terms and for KEGG pathways. The highest scoring enrichment category for each module is listed in Table 2. Each module can be divided into two subsets such that all correlations within a subset are positive (Methods). We also tested for enrichment within each of these subsets [Additional file 3: Supplemental Table S1]. Many of the module enrichment scores are highly significant indicating that correlated groups of variable genes are enriched for specific biological functions.

Most modules in a given tissue share similar features with at least one module in another tissue [Additional files 2, 4: Supplemental Figure S2; Additional file 5: Supplemental Table S2]. Several sets of modules shared similar patterns of between-mouse variation and had significant gene overlap and functional enrichment. Other sets of modules shared similar patterns of within-mouse variation, but with distinct between-mouse variation. Several pairs of modules had significant gene overlap but did not have correlated patterns of variation. Examples of each are described below.

### Between-mouse patterns of variation are shared across tissues

Modules from different tissues that are enriched for similar functional categories typically have high intraclass correlation and similar patterns of between-mouse variation. To quantify this similarity, we computed a between-mouse correlation, *r_b_*, for all pairs of module eigengenes by averaging the two within-mouse samples before computing the Pearson correlation.

Each of the four tissues has at least one module that is enriched for immune response. The heart-brown (*c *= 0.03), kidney-gold (*c *= 0.57), and liver-pink (*c *= 0.79) modules are enriched for the GO category *exogenous antigen presentation *(Table 2). The between-mouse correlations, *r_b_*, range from 0.53 to 0.80, and the genes in these modules overlap significantly based on a hypergeometric test (Methods). Pairwise overlaps range from 16 to 19 genes and seven genes (*Cd274*, *Cd74*, *Cxcl9*, *H2-DMa*, *H2-Eb1*, *Igtp *and *Iigp2*) are found in all 3 modules.

The kidney-blue (*c *= 0.42) and liver-brown (*c *= 0.74) modules are enriched for GO category *extracellular matrix*, each containing more than 12 genes of that category. Their between-mouse profiles are correlated (*r_b _*= 0.75) and they share 20 genes in common (out of 36) including *Adamts2*, *Col5a1*, *Col6a1*, *Col14a1*, *Ecm1*, *Igfbp3*, *Tgfbi *and *Timp2*.

The adipose-red (*c *= 0.42), heart-blue (*c *= 0.20), kidney-brown (*c *= 0.59) and liver-black (*c *= 0.78) modules are enriched for the GO category *apoptosis *and have between-mouse correlations, *r_b_*, ranging from 0.52 to 0.93. These modules overlap with 16 genes present in at least 3 of the 4 modules including *Ccrn4l*, *Gadd45g*, and *Map3k6*. The liver-blue module (*c *= 0.77) also has a high between-mouse correlation (*r_b _*≥ 0.64) and significant gene overlap with these adipose, heart and kidney modules including *Fkbp5 *and *Per1*.

The kidney-pink (*c *= 0.69) and liver-magenta (*c *= 0.74) modules have correlated between-mouse profiles (*r_b _*= 0.88), and each contains 18 or more genes of the GO category *DNA-dependent regulation of transcription*. Their gene overlap (12 out of 47) includes *Bcl6*, *Cish*, *Rgs3*, and *Socs2*.

The between-mouse profiles of the kidney-green (*c *= 0.59) and liver-red (*c *= 0.72) modules are correlated (*r_b _*= - 0.73) and each module contains 12 or more genes of the GO category *cellular lipid metabolic process*. They have 12 genes in common (out of 60) including *Acaa2*, *Acadm*, *Agxt2l1*, *Cyp26b1*, *Cyp4a10*, *Cyp4a14 *and *Slc2a2*.

### Within-mouse patterns are similar across modules of the same tissue

Some modules had similar patterns of within-mouse variation but different patterns of between-mouse variation. To measure similarity of within-mouse variation, we centred the sample values on individual mouse means and then computed a Pearson correlation, *r_w_*. This measure is only meaningful for comparisons within the same tissue as there is no correspondence between the duplicate samples from different tissues. Adipose and heart each have multiple highly correlated modules (|*r_w_*| ≥ 0.64). The adipose-green, adipose-red, adipose-black, and adipose-magenta modules have distinct patterns of between-mouse variation and different functional enrichment, but they all share high within-mouse correlation (Figure 4A, Table 2). A similar relationship was observed for the heart-green, heart-red, heart-turquoise, heart-blue, heart-brown, and heart-gold modules (Figure 4B, Table 2).

### Uncorrelated modules have gene overlap and similar functional enrichment

Some modules share genes and functional enrichment categories but do not have correlated patterns of variation. The adipose-gold (*c *= 0.12), heart-red (*c *= 0.00), and kidney-black (*c *= 0.10) modules have a high gene overlap (adipose-gold & heart-red, 48 out of 72; adipose-gold & kidney-black, 10 out of 29; heart-red & kidney-black, 25 out of 40 and 9 genes in all three including *Acaca*, *Cidea*, *Cox8b *and *Ucp1*). They are enriched for the GO category *fatty acid metabolic process*. The adipose-magenta (*c *= 0.00) and heart-gold modules (*c *= 0.00) share 118 out of 120 genes including *Cd8b1 *and *Lck *and are enriched for the GO category *immune system process*. The adipose-brown module (*c *= 0.07) shares 87 out of 182 genes with the heart-green module (*c *= 0.00) and 31 out of 35 genes with the liver-turquoise module (*c *= 0.02). These modules are enriched for the GO *actin cytoskeleton *category and share 8 genes in common including *Ckm *and *Myl1*. The adipose-turquoise (*c *= 0.44) and kidney-turquoise (*c *= 0.01) modules share 30 out of 40 genes including *Apoal*, *Cyp8b1*, and *Ugt2b3 *and are enriched for the KEGG pathway *complementation and coagulation cascades*. The adipose-green (*c *= 0.28) and heart-turquoise (*c *= 0.17) modules are overlapping in 12 out of 50 genes including *Ccl9*, *Cxcl1*, *Egr1*, *Fos*, and *Hmox1 *and are enriched for *chemokine activity*. The adipose-black (*c *= 0.00), heart-black (*c *= 0.00), kidney-magenta (*c *= 0.00), and liver-green (*c *= 0.39) modules have pairwise overlaps ranging from 33 to 146 genes. Twenty-two genes are shared among all 4 of these modules and they are enriched for KEGG pathway *oxidative phosphorylation *and the GO category *mitochondrial inner membrane*.

### Comparison across platform

We repeated the gene expression assays for only the liver samples on a different platform, the Affymetrix Whole-Transcript Mouse Gene 1.0 ST array. To facilitate comparison, we generated a cross-platform probe map based on gene annotation (Methods). Using this map, we computed eigengenes of the previously defined clusters from the Affymetrix data. Correlation of the eigengenes across platforms was very high for 7 of the 9 modules (*r *> 0.89 for 6 of 9 modules, *r *= 0.76 for liver-brown; [Additional files 2, 6: Supplemental Figure S3]). Two modules with lower correlation (liver-gold: *r *= 0.42, liver-green: *r *= 0.21) had less than 20% of variance explained by the eigengene with Affymetrix data. However, for the liver-gold module, low expression for mouse 6 was a consistent pattern across platforms. The profiles of all 19 genes that are highlighted in the Discussion (below) are highly correlated across platform (*r *> 0.55 for all 19, *r *> 0.70 for 16 of the 19, [Additional files 2, 7, 8, 9: Supplemental Figures S4-S6]).

## Discussion

There are several mechanisms that may contribute to between-mouse variation in gene expression in C57BL/6J mice. New mutations that create single nucleotide or copy number variants may result in variable gene expression. We expect such events to be rare. However, we have observed a striking pattern of differential expression (*r_b _*> 0.88; p < 0.01) in the insulin degrading enzyme (*Ide*) with approximately two-fold higher expression in all 4 tissues for the two mice of cage 4. We speculate that these siblings may have inherited a copy number variant at this locus on chromosome 19 for which copy number changes have been observed previously in C57BL/6J mice [12]. Genes that display circadian or other periodic expression patterns can be out of phase in different animals. We attempted to control for cyclical variation by collecting samples in a consistent and narrow time frame for all mice. Variation in feeding behaviour is another possible factor and although we implemented a 4-hour fast prior to tissue collection, some variation in time since last feeding is inevitable. Epigenetic differences may affect the expression of genes as a result of variable access to nutrients in utero, birth order, maternal stress or other pre- or post-partum events. Slight differences in phenotype at birth may be magnified over time. Response to subtle differences in local environment may have an effect on gene expression and finally, the expression of some genes may be sensitive to events just prior to euthanasia [3].

Within-mouse transcript variation could reflect stochastic variation in gene expression, which has been observed within individual cells and across cell populations [13-20]. However, if it is present, this effect seems to be dominated by other factors in our study. Tissue heterogeneity due, for example, to localization of stem and progenitor cell populations can result in sampling variation [21-24]. This variation may be amplified by dissection, especially in tissues with imprecise boundaries. Even a relatively homogenous and easily isolated tissue such as liver will have internal structure that can influence local gene expression [25,26].

### Phenotypic implications of between- and within-mouse variation in adipose tissue

**Figure 5:**
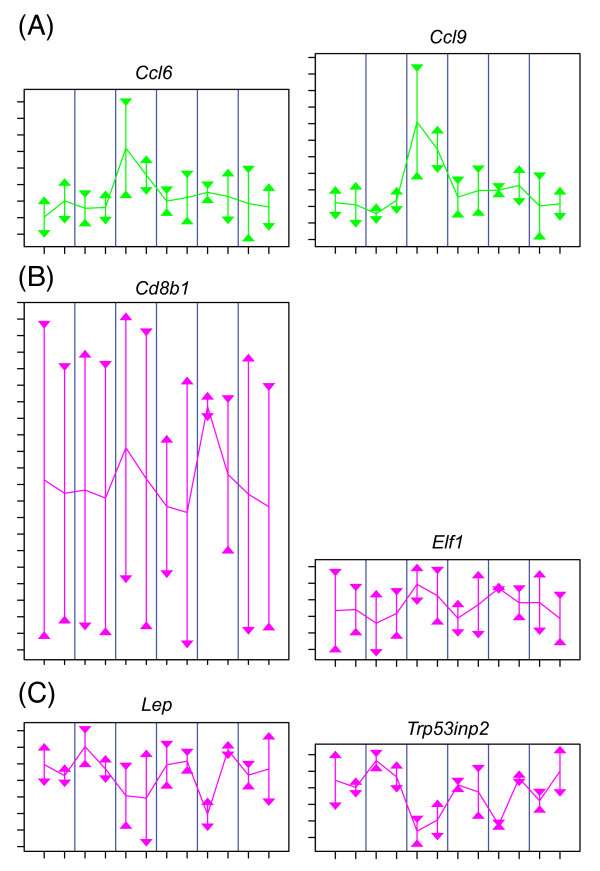
**Within-tissue correlation of eigengene transcript abundance profiles**. The eigengenes of the adipose-green and adipose-magenta modules exhibit negative within-mouse correlation and positive between-mouse correlation (*r_w _*= -0.65, *r_b _*= 0.69) Profiles are shown for (A) *Ccl6 *(l) and *Ccl9 *(r) of the positively correlated adipose-green module and KEGG *cytokine-cytokine receptor interaction *pathway, (B) *Cd8b1 *(l) and *Elf1 *(r) of the positively correlated adipose-magenta module and GO *immune response *biological process, and (C) *Lep *(l) and *Trp53inp2 *(r), of the negatively correlated adipose-magenta module. The graphical features of the eigengene plots of Figure 4 apply to these gene plots. Profiles are coloured by module membership. Summary statistics for these genes are available [Additional file 10: Supplemental Table S3].

Adipose tissue is compartmentalized into adipocytes, preadipocytes, and vascular epithelium [2]. The degree of vascularisation can vary significantly across different regions of the same fat pad and is expected to be greater in the portion of the inguinal fat pad that is near the inguinal lymph node [27]. Vascularised adipose tissue tends to be more metabolically active [28]. We found a large number of genes that have within-mouse variation related to vascularisation in the adipose-magenta module (1340 genes, *c *= 0.00). The positively correlated subset of this module is enriched for GO biological processes *immune response, T cell activation*, and *lymphocyte activation *[Additional file 3: Supplemental Table S1] and include genes expressed in lymphocytes such as *Lck*, *Cd8b1*, and *Elf1 *(Figure 5, [Additional file 10: Supplemental Table S3]). Some genes within the adipose-magenta module, which is dominated by within-mouse variation, also have large between-mouse fold changes. These genes, including *Bmp3*, *Sfrp5*, *Mest*, *Lep *and *Trp53inp2*, are positively correlated with body weight and were previously found to be predictive for adiposity [2] [Additional files 2, 11: Supplemental Figure S7]. They are also negatively correlated with the module eigengene, which is consistent with higher expression in the less vascularised region of the inguinal fat pad, suggesting an inverse relationship between vascularisation and adiposity.

We chose to study the inguinal fat pad because it can be efficiently dissected. Gene expression can vary among fat depots [29,30] and proximity to the inguinal lymph node clearly contributed to heterogeneity in the inguinal fat pad. This limits our ability to generalize our findings. However, our previous experience [31] indicates that other fat depots are at least as variable as the inguinal depot. The Koza et al. study [18] identified their adiposity signature, which we have replicated, in epididymal and retroperitoneal fat.

### Variable brown fat signature in white fat tissue

Several genes in the adipose-gold module are expressed exclusively in brown fat, including *Ucp1*, *Cidea*, and *Cox8b *[Additional files 2, 12: Supplemental Figure S8A-B]. This module is enriched for *fatty acid metabolism *and the module eigengene is correlated with *Prdm16 *(*r_b _*= 0.86; *r_w _*= 0.74; [Additional files 2, 12: Supplemental Figure S8C]), which is part of a transcriptional complex that promotes brown fat differentiation and suppresses skeletal muscle cell differentiation [32,33]. The adipose-brown module is enriched with 21 genes of the GO biological process *muscle contraction*. Genes in this module are expressed in both skeletal muscle and brown fat and many are related to brown fat cell differentiation [32,33]. We ruled out cross contamination with muscle tissue by inspection of the dissection procedure. The enrichment for *muscle contraction *appears to be spurious and reflects a potential pitfall of enrichment analysis using GO annotation.

Most of the variation in the adipose-gold (*c *= 0.12) and adipose-brown (*c *= 0.07) modules is attributable to the within-mouse component, which suggests a heterogeneous spatial distribution of brown fat within the inguinal fat pad. However, large between-mouse fold changes, including *Ckm*, with 56-fold change, the largest observed in this study [Additional files 2, 12: Supplemental Figure S8D], suggest that the proportion of brown fat may also vary across mice. Brown fat tissue proportion have previously been shown to vary with age, strain, and environmental conditions [34].

### Region-specific variation of gene expression in heart

The heart is composed primarily of cardiac smooth muscle, but it is differentiated into atrial, ventricular and trabecular regions with a left-right asymmetry. Several genes expressed in atria and trabeculae of the heart are repressed in the ventricles, in part, through activity of the transcription factor, *Gata4 *[35]. The heart-green module (898 genes) is enriched for these genes and shows a pattern of within-mouse variation with little between-mouse variation (*c *= 0.00). *Gata4 *is in the heart-red module (*c *= 0.00), which has a strong within-mouse correlation to the heart-green module (*r_w _*= 0.89) [Additional files 2, 13: Supplemental Figure S9]. *Gata4 *is negatively correlated with the heart-red eigengene such that the within-mouse variation in *Gata4 *is inversely related to the expression of ventricle-repressed transcripts [Additional files 2, 13: Supplemental Figure S9]. We compared our results with a study of chamber-specific gene expression (Tabibiazar et al. (2003) [36]) and found that, of the 27 genes previously reported to be more highly expressed in the atria than in the ventricles, 26 are included in the heart-green module. The relatively small magnitude of between-mouse variation in these modules reflects the effect of averaging of the two samples, which together comprise the whole heart. We conclude that much of the within-mouse variation observed for heart tissue is a consequence of variable proportions of anatomical substructures, specifically ventricular tissue, within the samples.

### Androgen-regulated genes are variable between mice in the kidney

Many genes are regulated in response to androgens. In mice, *Srd5a2 *plays a key role in androgen signal amplification [37] suggesting that androgenic effects in individuals with higher *Srd5a2 *expression could be more pronounced. *Hsd11b1 *facilitates the conversion of testosterone to adrenosterone [38] and has been shown to be androgen-responsive in mice [39]. These genes were found to be variable between mice and cluster together in the kidney-green module (*c *= 0.59), which is enriched for the KEGG *androgen and estrogen metabolism *pathway. Other androgen-responsive genes in the kidney-green module include *Cyp4a14*, *Slco1a1*, *Nudt19*, *Prlr*, *Angptl7*, *Hsd17b11*, and *Tmco3 *[Additional files 2, 14: Supplemental Figure S10].

It is not immediately clear if this variation reflects transient or steady state variation in androgen levels between mice. The expression of a mouse urinary protein, *Gusb*, is responsive to androgens in the long-term but not in the short-term [40]. *Gusb *has significant between-mouse variation that is correlated with the kidney-green module eigengene (*r_b _*= 0.73) (Figure 4, [Additional files 2, 14: Supplemental Figure S10]). This suggests that other genes in this module also reflect steady state androgen levels, which may have important physiological and behavioural implications.

### Between-mouse variation in fatty acid metabolism in the liver

Genes in the liver-red module have either low or high expression in the two mice of cage 3 [Additional files 2, 7: Supplemental Figure S4]. Genes in the low expression subset are involved in oxidation of fatty acids (*Acaa2*, *Acadm*, *Ces3*, *Crat*, *Cyp4a10*, *Cyp4a14*, and *Elovl3*). Genes in the high expression subset, specifically *Tnfrsf1a *and *Ptgis*, are involved in the conversion of the essential fatty acid arachidonic acid to prostaglandins. Thus, we see decreased fatty acid degradation in mice that are actively utilizing fatty acids. The liver-red module also shares genes with the androgen-associated kidney-green module which may reflect the requirement for lipids as precursors in androgen synthesis.

### Between-mouse variation in circadian rhythm

The adipose-red, heart-blue, kidney-brown, liver-blue, and liver-black modules are correlated and share multiple genes related to apoptotic activity, which varies following circadian rhythm in mice [41]. Several other genes that are known to vary in a circadian fashion are also found in these modules [Additional files 2, 8: Supplemental Figure S5], including *Ccrn4l*, *Fkbp5*, *Gadd45g*, *Map3k6*, *Per1*, *Pim3*, *Mt1*, *Sgk1*, *Errfi1*, *Cdkn1a*, *Dusp1*, and *Angptl4*. The core circadian gene *Per2 *[42,43] is found in the adipose-red module. Genes that follow a circadian expression pattern are expected to vary with the time of day and with fasting/feeding cycles. Despite our efforts to control both of these factors, between-mouse variation can be expected to arise if the mice are in slightly different phases of their diurnal cycles.

*Angptl4*, *Cdkn1a*, *Dusp1*, and *Fkbp5 *vary in circadian fashion [43,44] and are all located in a 7 Mb region on proximal chromosome 17. This region is the strongest example of coexpression clustering that we found in this study. However, statistical assessment suggests that a cluster of this size could be explained by chance.

### Between-mouse variation associated with growth hormone

The genes *Socs2*, *Bcl6*, *Cish*, and *Gadd45g *have correlated patterns of variation in kidney and liver and are among the genes with the most significant between-mouse variation [Additional files 2, 9: Supplemental Figure S6]. Growth hormone has been shown to elicit a strong transcriptional response in *Socs2 *(positive), *Cish *(positive), *Bcl6 *(negative), and *Gadd45g *(positive), as well as in the growth hormone responders *Igf1 *and *Il6 *[45]. Three of these genes (*Socs2*, *Bcl6*, and *Cish*) belong to the kidney-pink and liver-magenta modules, which have 12 overlapping genes and are enriched for genes involved in transcription regulation. Growth hormone signalling affects transcription of genes such as *Xbp1 *(kidney-pink, liver-magenta), which is critical for the regulation of hepatic lipogenesis [46]. The effect of growth hormone signalling appears to extend beyond these modules, however. Among 71 genes previously identified as growth hormone responders [47], 49 were classified as variable in our study, indicative of widespread individual variation in growth hormone signalling.

### Similarities and differences in transcript abundance for sibling cage mates

Sibling cage mates may be expected to exhibit greater similarity than randomly selected mice of the same strain due to shared developmental or micro-environmental factors. When we further partitioned the between mouse variance into between-cage and within-cage components (Methods), we found more genes with significant between-cage variation (adipose, 318; heart, 294; kidney, 1003; liver, 2066) than within-cage variation (adipose, 91; heart, 77; kidney, 639; liver, 1652). The liver-red module provides a striking example of within-cage similarity [Additional files 2, 7: Supplemental Figure S4]. Enrichment for genes associated with fatty acid oxidation in this module could reflect an extended period of fasting just prior to euthanasia. For example, expression of murine hepatic *Cyp4a14 *(liver-red module) is known to increase in expression under fasting conditions [48]. This gene has been reported to be variable between strains in liver [4], but it is not clear whether this is a genuine strain-specific effect or an artefact due to co-housing of mice of the same strain.

Other factors could contribute to greater differences between mice within a cage. Cohabitating outbred male mice form a social structure that includes dominance status even when mice are housed as siblings from birth. Dominance behaviour has been observed within male mice of some inbred strains (e.g. CBA, DBA2) but not C57BL/6J. However, cohabitation has known phenotypic effects on C57BL/6J males including change in body weight, adrenal weight, and aggressiveness [49-53]. The factors that determine the social status of siblings raised together are unclear, but once established, social behaviour can reinforce these minor differences leading to distinct individual phenotypes in adult mice.

In our experiment, we observed within-cage body weight difference of as much as 3g (10% of total body weight). Some of the transcriptional changes that we have observed are likely to be related to these body weight differences. For example, in cage 5 we observed a large body weight difference coincident with a large difference in transcription of signature genes for adiposity [Additional files 2, 11: Supplemental Figure S7], but small differences in signature genes for androgen levels [Additional files 2, 14: Supplemental Figure S10]. In contrast, in cages 3 and 4, body weight differences coincide with a transcriptional signature for androgen response but not for adiposity. This suggests that bodyweight differences may reflect two distinct processes, one that affects adiposity and another that affects androgen levels and lean mass [54,55]. Moreover, these findings provide evidence for an effect of social context on biological processes that have important consequences for human health.

### Comparison to a previous study of transcript variation

We directly compared our results to a previous study of transcriptional variation in C57BL/6J mice [3,56] by computing variance components and applying the same significance tests to both data sets [Additional file 15: Supplemental Table S4]. We found little correlation in total variation (*r *< 0.09) which we attribute to the predominance of technical variation, especially in the older study. However, we did find good agreement across these studies when we examined specific genes highlighted in the previous study. *Cfd *was reported to vary significantly between mice in the kidney for the previous experiment in which effects due to dissection and RNA extraction are included in the between-mouse variance component. We also found it to be a variable gene, but, in contrast, we identified *Cfd *as a gene with primarily within-mouse variation (*c *= 0.11) in the kidney-black module [Additional files 2, 16: Supplemental Figure S11]. Both studies identified significant between-mouse variation in several highly variable genes, including *Gadd45g*, *Dusp1, Cish*, and *Bcl6 *[Additional files 2, 8, 9: Supplemental Figures S5-S6]. Our study, with a larger sample size, a more recent array technology, and a different experimental design should provide a more precise and detailed picture of variation in gene expression.

## Conclusions

Transcript abundance varies significantly among genetically identical male C57BL/6J mice housed under uniform conditions. Patterns of variation can be tissue specific or shared across multiple tissues and transcripts can vary between tissue samples collected from the same animal. Groups of genes with correlated patterns of between-animal or within-animal variation are often enriched for specific functional annotations. We utilized correlation-based clustering to organize a large number of distinct patterns of variation. Literature search tools and functional annotation aided in the interpretation of our findings. However, annotation of gene function is incomplete and this presented some challenges as exemplified by the finding of a skeletal muscle signature in white adipose tissue, which was due to the presence of a related cell type, brown fat.

This study highlights a number of potential biological and technical sources of variation that practitioners should be aware of for both experimental design and interpretation. Much of the between-animal variation reported here reflects functions that are sensitive to environmental cues, such as androgen response, circadian rhythm, and immune response. External environmental cues tend to elicit similar responses in multiple tissues. Variation of gene expression within tissues reflects their heterogeneous cellular composition, and is also a major factor contributing to variation in gene expression. This underscores the potential for dissection or biopsy procedures to introduce unwanted variation into studies of gene expression. Adipose tissue is especially problematic in this regard as it is a highly dynamic and heterogeneous tissue with few anatomical features to guide consistent dissection.

Our tissue collection procedure involved a coarse separation of tissue fragments which, in retrospect, was useful to reveal within-tissue heterogeneity. An exception to this was our use of the intact left and right kidneys as replicates. This may explain the relatively low within-mouse variation observed for this heterogeneous and highly structured tissue. In future studies, we recommend the use of procedures that more effectively homogenize tissue, such as pulverization and mixing of snap frozen samples. Our finding also raises questions about the potential for introducing systematic variation in the dissection of anatomical substructures. This may be a particular concern for studies of gene expression in the brain, for which we have no data at this time.

The presence of biologically meaningful covariation in a setting with no experimental perturbation underscores the need for replication and careful adherence to statistical design principles in gene expression studies. Seemingly innocuous experimental factors such as co-housing of mice can result in systemic differences that may lead to strong statistical support for incorrect conclusions. Prior knowledge of the categories of genes that are intrinsically variable can help to identify such effects. Our study further demonstrates that the variation used to construct statistical tests (error variance) in microarray experiments can have substantial correlation across large sets of genes. This can have a profound impact on testing procedures, especially those that rely on multiple test adjustment of p-values across many genes [57].

## Methods

### Animals and RNA isolation

We obtained 12 C57BL/6J male mice from The Jackson Laboratory. Six pairs of littermates were housed together from weaning and put on LabDiet's 5k52 diet (standard chow containing 6% fat) in a facility with a 12 h:12 h light:dark cycle beginning with lights on at 6:00 a.m. Animals had *ad libitum *access to food and acidified water. At 10 weeks of age, body weight was recorded and the mice were euthanized by cervical dislocation and perfused with RNase-free DEPC-treated PBS. Dissection procedures were started at 11:00 a.m. after a 4-hour period of food deprivation and were completed within a one-hour time window. The Jackson Laboratory Animal Care and Use Committee approved the animal housing and experimental procedures described in this work. Inguinal fat pad, heart, liver, and both kidneys were dissected, cut into pieces not exceeding 0.5 cm in any dimension, divided into two samples and placed in 15 ml conical tubes containing RNAlater solution (Ambion, Austin TX). Each kidney sample consisted of one complete kidney, left or right. Tissues were homogenized in TRIzol™ reagent (Invitrogen, Carlsbad, CA). Total RNA was isolated by standard TRIzol™ methods according to the manufacturer's protocols, and quality was assessed using an Agilent 2100 Bioanalyzer instrument and a RNA 6000 Nano LabChip assay (Agilent Technologies, Santa Clara, CA). The RNA was treated with DNase1 (Qiagen, Valencia, Ca.) according to the manufacturer's methods.

### Microarray processing

#### Illumina Sentrix^® ^Mouse-6 v1.1 BeadChip processing

Total RNA was reverse transcribed followed by second strand cDNA synthesis. For each sample, an in-vitro transcription (IVT) reaction was carried out incorporating biotinylated nucleotides according to the manufacturer's protocol for Illumina^® ^Totalprep RNA amplification kit (Ambion). 1.5 μg biotin-labelled cRNA was then hybridized onto Mouse-6 Expression BeadChips (Illumina, San Diego CA) for 16 hours at 55°C. Post-hybridization staining and washing were performed according to manufacturer's protocols (Illumina). Illumina Sentrix^® ^Mouse-6 v1.1 BeadChips were scanned using Illumina's BeadStation 500 scanner. Images were checked for grid alignment and then quantified using the BeadStudio software. Control summary graphs generated by BeadStudio were used as quality assurance tools for hybridization, washing stringency, and background. Integrity of the arrays was investigated using the BeadStudio array images and also using bead level image plots generated using the R/beadarray package. Mean pixel intensities by bead type, were created using BeadStudio v3.1 and processed with the R/beadarray package [58]. We performed the experiment in two blocks of three cages, separated by one month. Within each block, we assayed gene expression in each tissue (12 samples) using two Illumina Sentrix^® ^Mouse-6 v1.1 BeadChips. Samples were randomly assigned to array positions within each chip with the constraint that samples from the same mouse were placed on separate chips. Quantile normalization [59] was applied within each tissue, and a correction for batch effects was applied separately for each gene using an MM-regression estimator from the R/robustbase software package [60]. We selected 45905 probes which are mapped to 22869 genes based on the R/illuminaMousev1p1BeadID.db package [61]. A transcript was called expressed if the mean intensity across the 2 samples of (at least) 1 mouse was above the 95th percentile of the distribution of the mean intensities for the negative control probes. The data are available in accession series GSE20121 from the Gene Expression Omnibus http://www.ncbi.nlm.nih.gov/geo/.

#### Affymetrix Mouse Gene 1.0 ST Array processing

Following reverse transcription with random T7 primers (Affymetrix, Santa Clara, CA), double stranded cDNA was synthesized with the GeneChip^® ^WT cDNA Synthesis and Amplification Kit (Affymetrix, Santa Clara, CA). In an in vitro transcription (IVT) reaction with T7 RNA polymerase, the cDNA was linearly amplified to generate cRNA. In the second cycle of cDNA synthesis, random primers are used to generate single stranded DNA in the sense orientation. Incorporation of dUTP in the cDNA synthesis step allows for the fragmentation of the cDNA strand utilizing uracil DNA glycosylase (UDG) and apurinic/apyrimidinic endonuclease 1 (APE 1) that specifically recognizes the dUTP and allows for breakage at these residues. Labeling occurs by terminal deoxynucleotidyl transferase (TdT), where biotin is added by an Affymetrix Labeling Reagent. 2.3 μg of biotin-labeled and fragmented cDNA was then hybridized onto GeneChip^® ^Mouse Gene 1.0 ST Arrays (Affymetrix) for 16 hours at 45°C. Post-hybridization staining and washing were performed according to manufacturer's protocols using the Fluidics Station 450 instrument (Affymetrix). Then, the arrays were scanned with a GeneChip™Scanner 3000 laser confocal slide scanner, quantified, and exported to .CEL file format using the GeneChip^® ^Operating Software. Probes were mapped to 34760 probe sets using the R/mogene10stv1.r3cdf package. The .CEL files were processed using the R/affy package using the Robust Multichip Average normalization method [59]. The probe sets were mapped to genes using the R/mogene10sttranscriptcluster.db package [62]. For this experiment, we used a partially balanced incomplete block design method that accommodated hybridization and washing/staining batch factors. Data are available as part of accession series GSE20121 from the Gene Expression Omnibus http://www.ncbi.nlm.nih.gov/geo/.

### Identifying variable genes and estimating variance components

For gene *g *= *1*,...,*G*; mouse *i *= *1,2,3,...,12*; and sample within mouse *k *= *1,2*, we assumed that the log-transformed transcript abundance, *y_ikg,_*, satisfies *y_ikg _*~ N(*0*, *σ_g_^2^*) and considered the null hypothesis H_0_: *σ_g_^2 ^*= *σ^2 ^*where *σ^2 ^*is a fixed variance common to all genes. The alternative is that some genes, *g*, have excess variability: H_a_: *σ_g_^2 ^*>*σ^2^*. To test this hypothesis, we compared the observed distributions of variance to a χ^2 ^distribution for each tissue. The distributions were scaled by dividing each variance by the robust bias-corrected James-Stein estimate [63]. For each tissue, the frequency of genes in the tail of the scaled distribution was then compared to the frequency of a random sample from a χ^2^*_23 _*distribution. We identified 2000-4000 genes in each tissue with greater than expected variance (*α *= 0.05) (Table 1). The 2500 most variable genes in each tissue were designated as *variable genes *and were used in the coexpression network analysis. We chose this number of genes due, in part, to computational constraints of the coexpression network analysis.

We used random effects ANOVA to decompose total variance into between-mouse and within-mouse variance components. Briefly, each *y_ikg _*is written as the sum of the average transcript abundance for that gene, *μ_g_*, a mouse-specific effect, *b_ig_*, and a within-mouse term, *w_ikg_*.(1)

The within-mouse term absorbs variation from the mean not accounted for by other terms on the right side of (1). The terms *b_ig_*, and *w_ikg _*are assumed to satisfy *b_ig _*~ N(*0*, *σ*_b*g*_*^2^*) and *w_ikg _*~ N(*0*, *σ_wg_^2^*), respectively. The terms *σ_bg_^2 ^*and *σ_wg_^2 ^*are the between-mouse and within-mouse variance components in this model. Estimates, *s_bg_^2 ^*and *s_wg_^2^*, for these components were obtained by residual maximum likelihood (REML) estimation from R/lme4 [64]. A modified F statistic [65] was used to identify transcripts with significant between-mouse variation. False positive rates were estimated using p-values that were calculated by permuting model residuals. Two types of multiple test corrections were performed. The p-values were adjusted using the Sidak step-down approach [5], and the Benjamini and Hochberg method [6]. The qvalue software package [7] was used to estimate the number of genes that do not have significant between-mouse transcript variation, *π_0_*. To separately assess significance of between-cage and within-cage variation, the following model was used: Each *y_ikg _*is written as the sum of the average transcript abundance for that gene, *μ_g_*, a cage-specific effect, *c_ig_*, a mouse-within-cage term, *d_j(i)g_*, and a within-mouse term, *w_ikg_*.(2)

The Pritchard et al. (2001) [3] data were revised to correct a processing error as previously reported [56]. For comparative purposes, we applied the same tests for significance of between-mouse variation described above to the corrected data.

### Coexpression network analysis

Variable genes were analysed separately for each tissue using coexpression networks [8,9]. Every pair of genes was given a weighted connection, *r_s_*^2^, equal to the square of their correlation coefficient across all samples. Transcript abundance profiles were hierarchically clustered and modules were obtained by a dynamic dendrogram cutting method and subsequent module merge procedure [66]. We only retained modules with more than 25 members. Modules are referenced by their tissue of origin and by a colour index.

For each module, the first principal component was computed to give a representative profile, referred to as the module eigengene [10]. We determined the sign of the module eigengene to be positively correlated with the majority of genes in the module and refer to this majority as the positively-correlated module genes. The complementary genes are referred to as the negatively-correlated module genes. Module eigengenes were scaled to match the median variance over all genes in the module (Figure 4). For each gene, we computed the intraclass correlation coefficient, *c *≡ *s_b_^2^/*(*s_w_^2 ^+ s_b_^2^*) as a measure of the relative contribution of the between-mouse variance component. We decomposed each gene profile into a between-mouse profile and a within-mouse profile. The between-mouse profile averages the two samples within each mouse and the within-mouse profile is the difference between sample 1 and the average value for that mouse. To measure similarity of between- and within-mouse profiles, we computed Pearson correlation coefficients, *r_b _*and *r_w_*, for between-mouse (*r_b_*) and within-mouse (*r_w_*) profiles. When assessing significance of similarity of correlation among eigengenes [Additional file 5: Supplemental Table S2], we applied a Fisher transformation with sample size *n *= 11 (*r_b_*) and *n *= 12 (*r_w_*). For significance *α *< 0.05, this required |*r_b_*| > 0.66 and |*r_w_*| > 0.64.

### Gene set enrichment

Each module of the coexpression networks was tested for enrichment within the Gene Ontology (GO) gene sets [67] and the Kyoto Encyclopaedia of Genes and Genomes (KEGG) pathway gene sets [38,68,69]. The universe was defined as the set of variable genes present in the relevant database (either GO or KEGG). Only one probe per gene was included in the set of variable genes. The positively- and negatively-correlated subsets of each module were also tested for enrichment. We considered two modules to have significant overlap of functional enrichment if there were 4 genes in each module from a given category and enrichment p-values were less than p < 0.01 for the category in all modules.

### Module overlap

We tested for overlap of modules across tissues on a pairwise basis using the hypergeometric test with a Bonferroni multiple-testing correction (*α *< 0.05). We also used the hypergeometric test to assess the significance of the overlap between published gene lists and modules in our study. In this case, the universe of genes was defined as those assayed in our experiment.

### Across-experiment comparison

To compare the results of the replicated liver experiments, a map from Illumina probe to Affymetrix probe-set was created based on gene symbol annotation. Where multiple Affymetrix probe sets had the same gene symbol assignment, we selected the one with highest correlation to the Illumina probe. For Affymetrix module eigengene calculation, we excluded Affymetrix probe sets with average intensity less than 7.

To compare our variance component distributions with those of Pritchard et al. (2001), we generated a map from Illumina probe to gene symbol annotation for the Pritchard et al. experiment [70]. Where multiple probe sets had the same gene symbol assignment, we selected the one with highest intraclass correlation coefficient. For this selection and for our comparison of total variation, we excluded the array component of variation for the Pritchard et al. experiment.

### Additional resource: Database

An on-line resource has been created to allow access to the experimental data, graphics, and test statistics for all probes in this study:

http://cgd.jax.org/individualvariation.shtml.

## Authors' contributions

KLS and GAC conceived the experiment. KLS directed the animal husbandry and tissue collection. GAC and PTV designed and implemented microarray experiments. PTV performed the data analysis. PTV and GAC wrote the manuscript. All authors read and approved the final manuscript.

## Supplementary Material

Additional file 1 **Supplemental Figure S1**. P-value histograms for between-mouse significance tests. Click here for file

Additional file 2 **Supplemental Figure Captions**. Captions for supplemental figures. Click here for file

Additional file 3 **Supplemental Table S1**. Enrichments scores of the gene coexpression modules. Click here for file

Additional file 4 **Supplemental Figure S2**. Graphical model showing relationships between modules. Click here for file

Additional file 5 **Supplemental Table S2**. Pairwise Pearson correlations for module eigengenes. Click here for file

Additional file 6 **Supplemental Figure S3**. Cross-platform comparison of liver eigengene profiles. Click here for file

Additional file 7 **Supplemental Figure S4**. Transcript abundance profiles for fatty acid metabolism genes in the liver. Click here for file

Additional file 8 **Supplemental Figure S5**. Transcript abundance profiles for circadian rhythm genes. Click here for file

Additional file 9 **Supplemental Figure S6**. Transcript abundance profiles for growth-hormone regulated genes in kidney and liver. Click here for file

Additional file 10 **Supplemental Table S3**. Within-mouse correlation statistics for selected genes in adipose. Click here for file

Additional file 11 **Supplemental Figure S7**. Transcript abundance profiles for variable genes reported in adipose tissue. Click here for file

Additional file 12 **Supplemental Figure S8**. Transcript abundance profiles for variable brown fat signature genes in white fat tissue. Click here for file

Additional file 13 **Supplemental Figure S9**. Transcript abundance profiles showing region-specific variation of gene expression in heart. Click here for file

Additional file 14 **Supplemental Figure S10**. Transcript abundance profiles for androgen-regulated variable genes in the kidney. Click here for file

Additional file 15 **Supplemental Table S4**. Transcript abundance variation statistics for Pritchard et al (2001) dataset. Click here for file

Additional file 16 **Supplemental Figure S11**. Transcript abundance profile for *Cfd *gene in kidney. Click here for file
