# Supplementary figures and images for: Stochastic variation of transcript abundance in C57BL/6J mice
Source: BMC Genomics. 2011 Mar 30;12:167. doi: 10.1186/1471-2164-12-167 (PMC3082245; doi:10.1186/1471-2164-12-167)

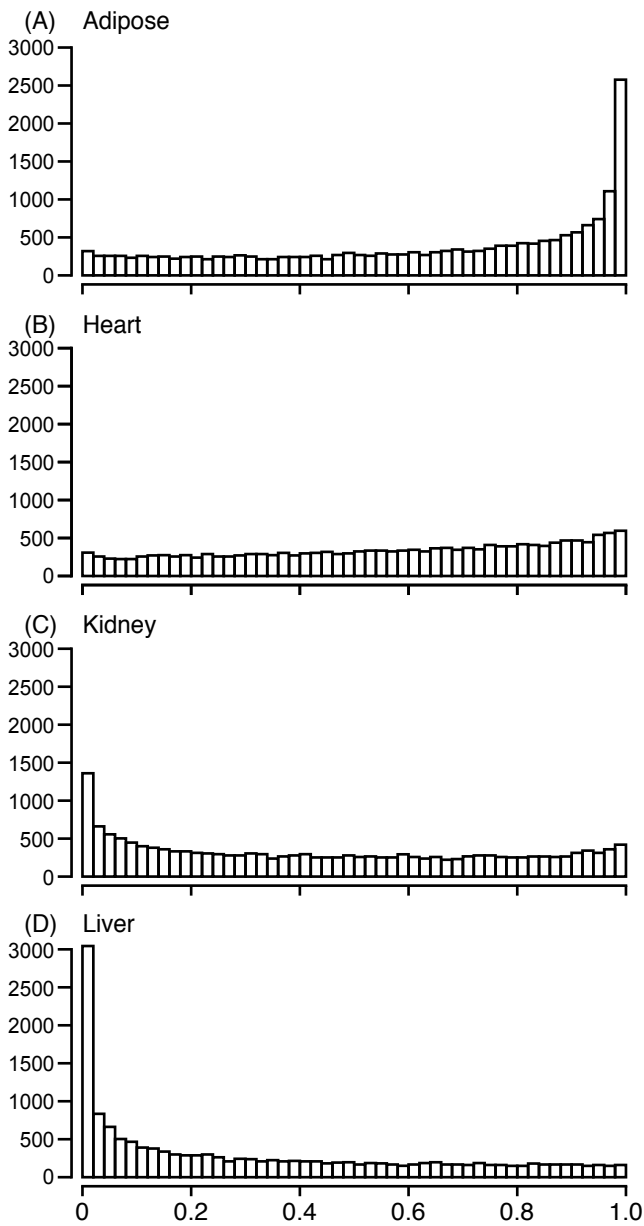

Supplement: Additional file 1 — Supplemental Figure S1. P-value histograms for between-mouse significance tests. [file 1471-2164-12-167-S1.PDF]

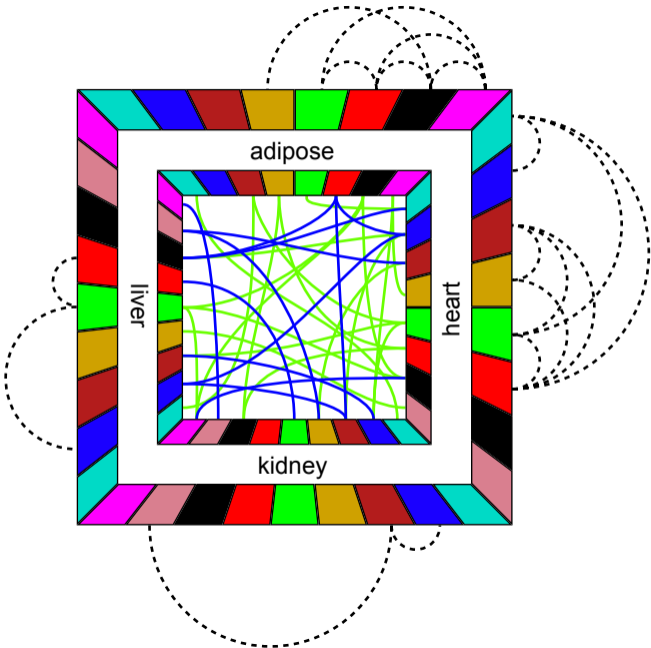

Supplement: Additional file 4 — Supplemental Figure S2. Graphical model showing relationships between modules. [file 1471-2164-12-167-S4.PDF]

(A) Liver Illumina

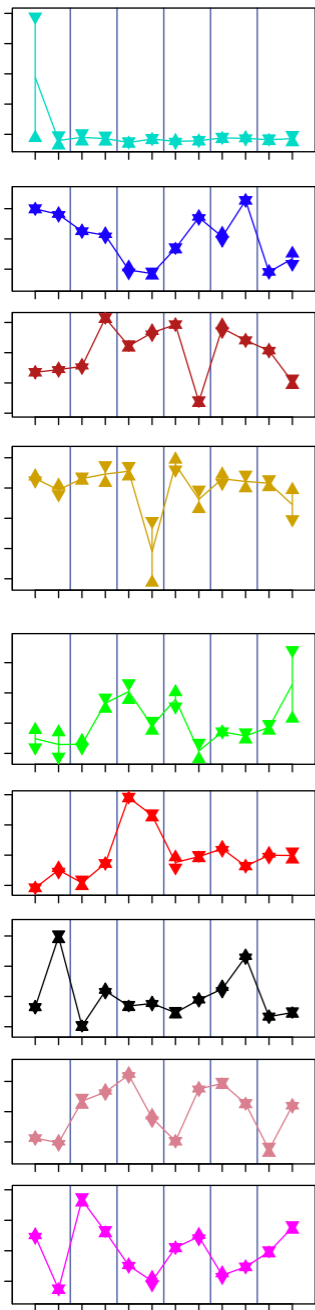

(B) Liver Affymetrix

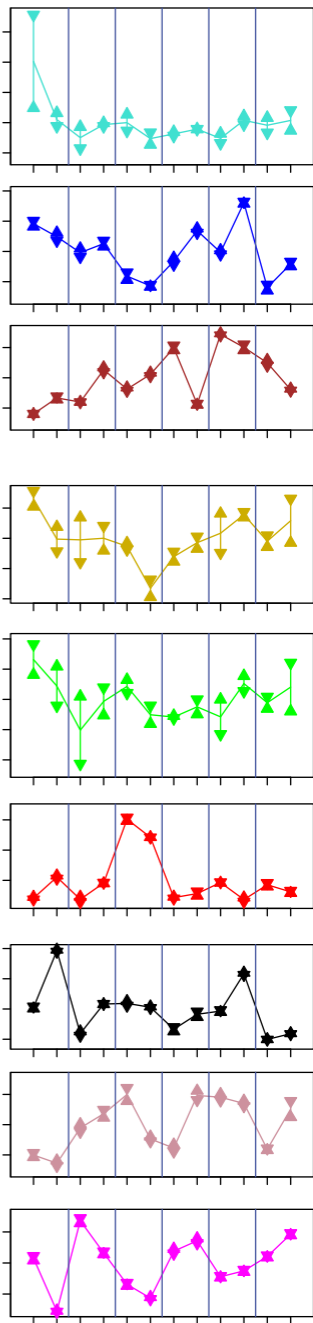

Supplement: Additional file 6 — Supplemental Figure S3. Cross-platform comparison of liver eigengene profiles. [file 1471-2164-12-167-S6.PDF]

**A**

Illumina

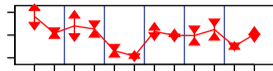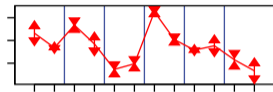

Supplement: Additional file 7 — Supplemental Figure S4. Transcript abundance profiles for fatty acid metabolism genes in the liver. [file 1471-2164-12-167-S7.PDF]

(E) Liver Affymetrix

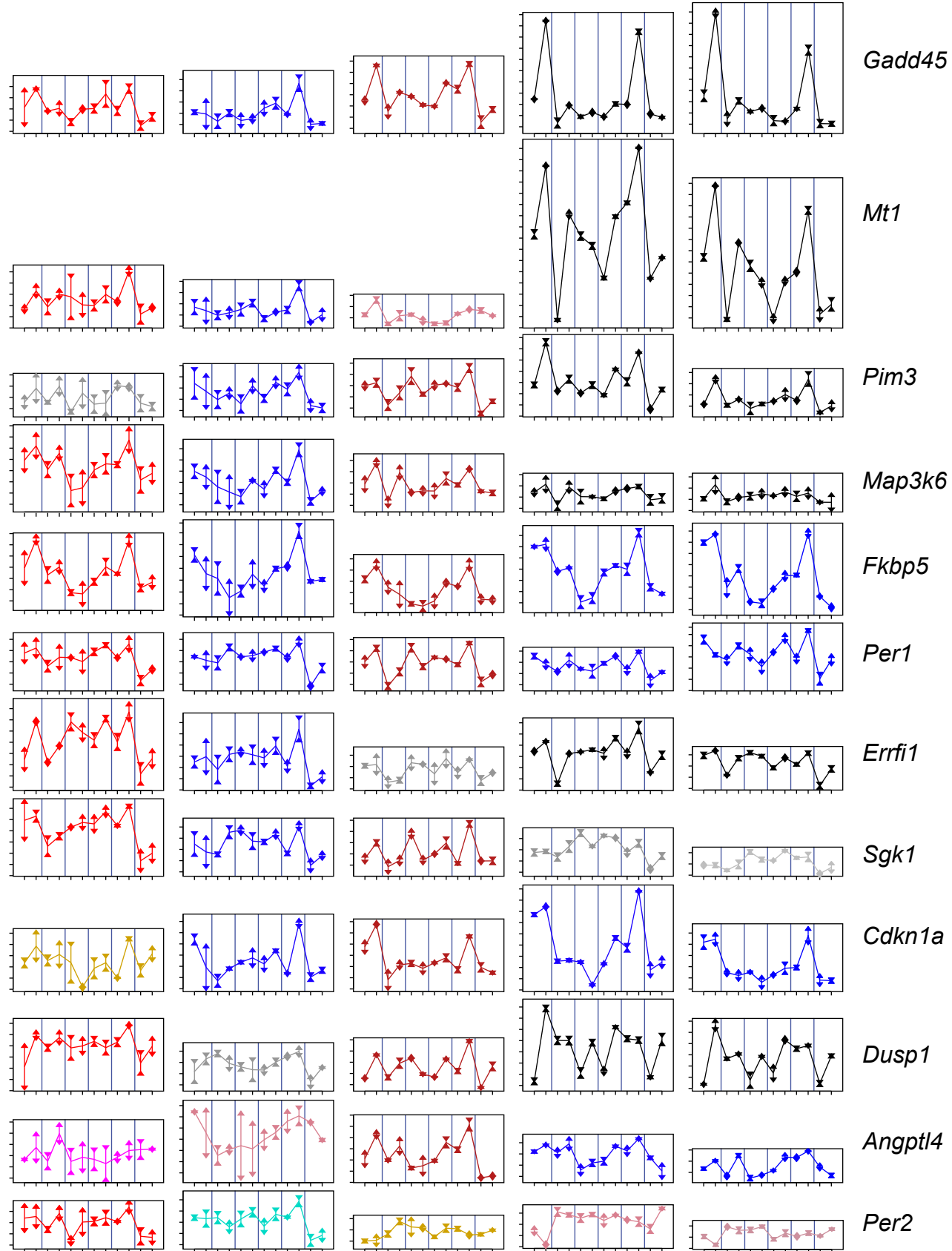

Supplement: Additional file 8 — Supplemental Figure S5. Transcript abundance profiles for circadian rhythm genes. [file 1471-2164-12-167-S8.PDF]

(A) Kidney

(B) Liver Illumina

(C) Liver Affymetrix

*Socs2*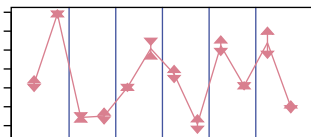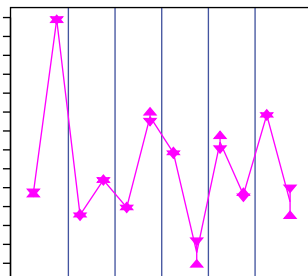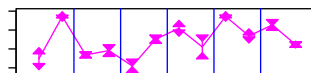*Gadd45g*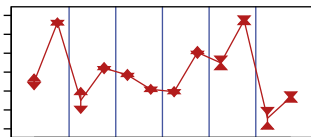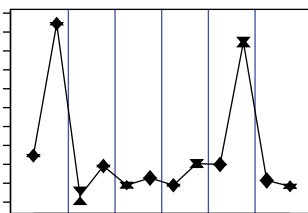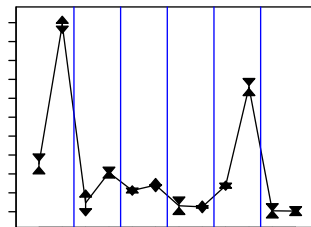*Cish*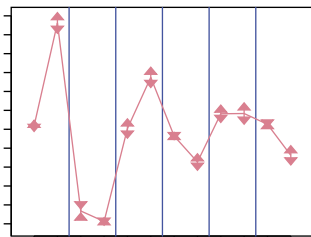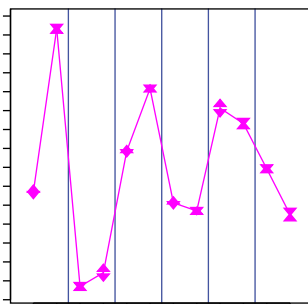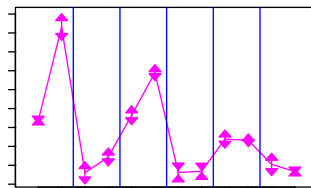*Bcl6*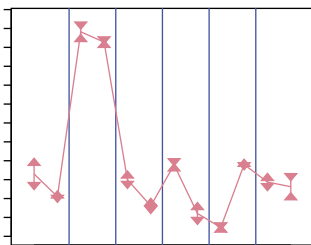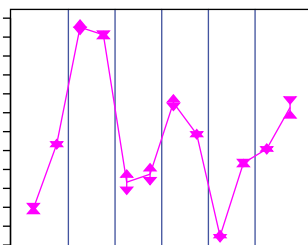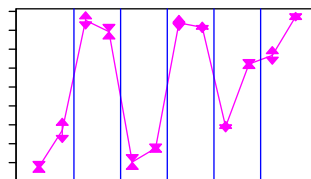

Supplement: Additional file 9 — Supplemental Figure S6. Transcript abundance profiles for growth-hormone regulated genes in kidney and liver. [file 1471-2164-12-167-S9.PDF]

(A)

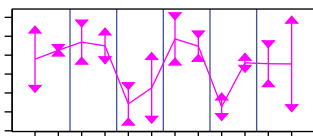

*Sfrp5*

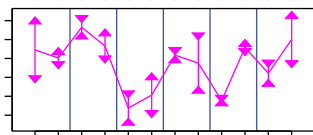

*Trp53inp2*

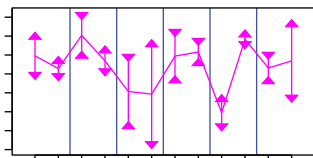

*Lep*

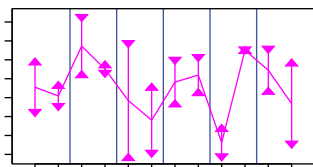

*Mest*

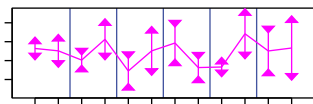

*Bmp3*

(B)

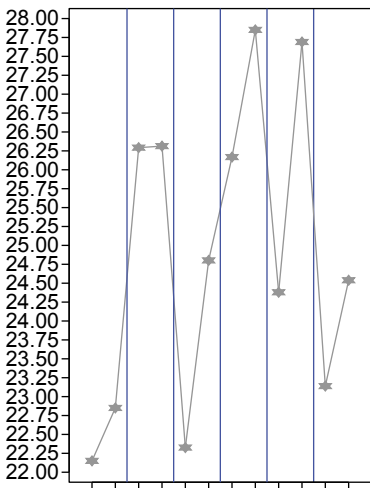

Body Weight

Supplement: Additional file 11 — Supplemental Figure S7. Transcript abundance profiles for variable genes reported in adipose tissue. [file 1471-2164-12-167-S11.PDF]

(A) *Ucp1*

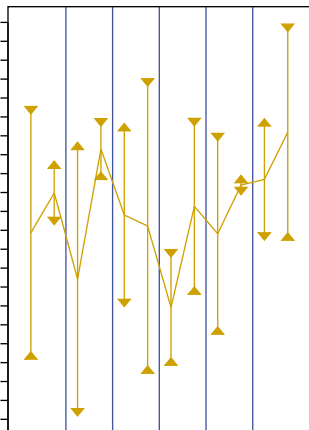

(B) *Cidea*

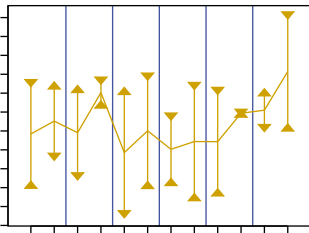

(C) *Prdm16*

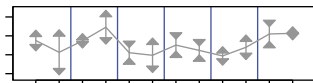

(D) *Ckm*

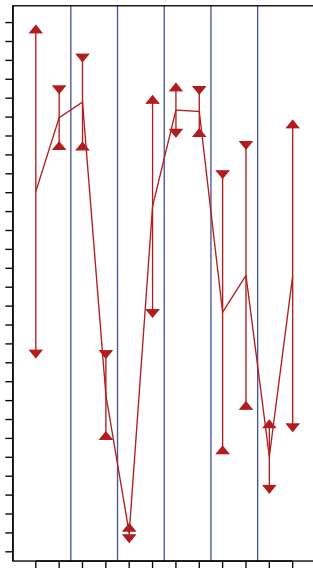

Supplement: Additional file 12 — Supplemental Figure S8. Transcript abundance profiles for variable brown fat signature genes in white fat tissue. [file 1471-2164-12-167-S12.PDF]

(A)

*Myl4*

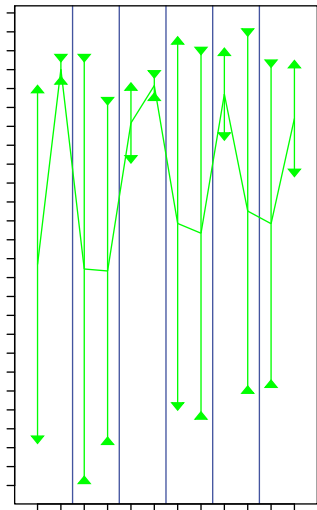

*Tbx5*

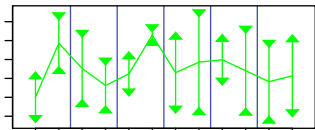

*Gja5*

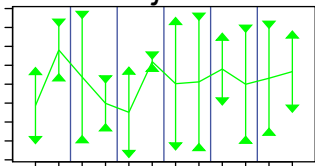

*Bmp10*

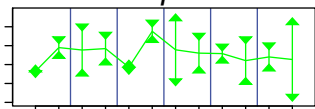

*Myl7*

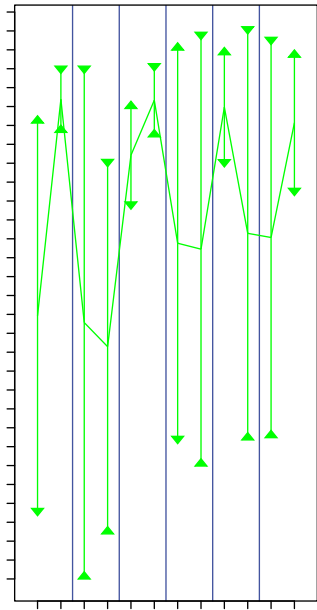

(B)

*Gata4*

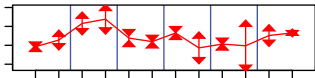

Supplement: Additional file 13 — Supplemental Figure S9. Transcript abundance profiles showing region-specific variation of gene expression in heart. [file 1471-2164-12-167-S13.PDF]

*Hsd11b1*

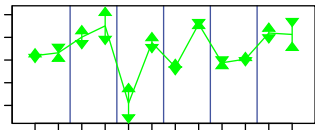

*Hsd17b11*

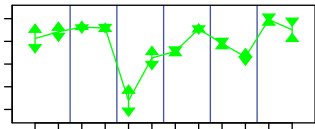

*Srd5a2*

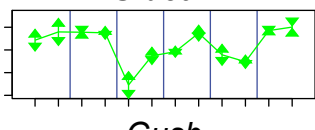

*Gusb*

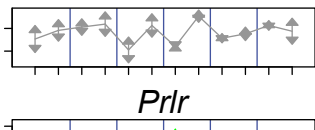

*Prlr*

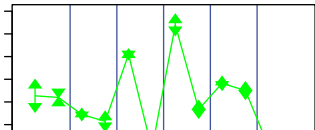

Supplement: Additional file 14 — Supplemental Figure S10. Transcript abundance profiles for androgen-regulated variable genes in the kidney. [file 1471-2164-12-167-S14.PDF]

*Cfd*

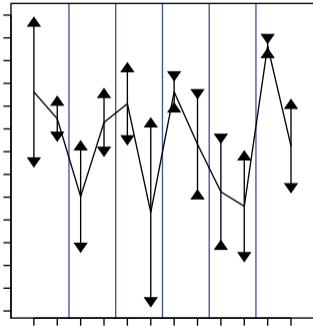

Supplement: Additional file 16 — Supplemental Figure S11. Transcript abundance profile for Cfd gene in kidney. [file 1471-2164-12-167-S16.PDF]
