# Supplementary material for: Stochastic variation of transcript abundance in C57BL/6J mice
Source: BMC Genomics. 2011 Mar 30;12:167. doi: 10.1186/1471-2164-12-167 (PMC3082245; doi:10.1186/1471-2164-12-167)
Supplement: Additional file 2 — Supplemental Figure Captions. Captions for supplemental figures. [file 1471-2164-12-167-S2.DOC]

# Supplemental Figure Captions

**Supplemental Figure S1: P-value histograms for between-mouse significance tests**

The frequency distributions of p-values for the *F_s_* test of between-mouse variance are shown for expressed genes by tissue. The histograms for kidney and liver have modes with p<0.05, indicating that there are more genes with small p-values than expected by chance . For adipose and heart, the p-value histogram may have a small mode near p = 0, but there are unexpectedly large modes near p = 1. In adipose, there are 1003 genes with p > 0.99 compared to 110 expected by chance. Of these, 476 genes were classified as variable genes and all are in the adipose-magenta module. They have highly correlated profiles with little between-mouse variation (*s _b_* = 0, *c*= 0.00 for all) and large within-mouse variation (median of *s _w_* = 0.31). Thus, the excess of p-values near 1 is due to a large block of genes that share the same pattern of variation.

**Supplemental Figure S2: Graphical model showing relationships between modules**

A graphical illustration of the relationships between modules is given. The modules of each tissue are represented by colour strips on the borders of the rectangle as indicated. The dashed external edges indicate modules that have between- or within-mouse correlation in the same tissue. The solid green and solid blue internal edges indicate cross-tissue pairs of modules with gene overlap. The module pairs with solid blue internal edges also exhibit between-mouse correlation of eigengenes.

**Supplemental Figure S3: Cross-platform comparison of liver eigengene profiles**

The liver eigengene transcript abundance profiles for the Affymetrix data are shown. The graphical features of the eigengene plots of Figure 4 apply to these plots. The Illumina liver eigengene profiles of Figure 4 are reproduced in the left panel here for ease of comparison. The profiles exhibit cross-platform correlation that satisfies *r* > 0.76 except for the liver-gold (*r* > 0.42) and liver-green (*r* > 0.21) modules.

**Supplemental Figure S4: Transcript abundance profiles for fatty acid metabolism genes in the liver**

The liver transcript abundance profiles for (from top to bottom) the KEGG pathway *fatty-acid metabolism* genes, *Acadm*, *Crat*, and *Cyp4a14*, and the GO *prostaglandin synthesis* gene, *Tnfrsf1a,* are shown. The graphical features of the eigengene plots of Figure 4 apply to these gene plots. For each gene, the probe identifiers, intraclass correlation coefficient (*c*), *F_s_* p-value, between-mouse Pearson correlation coefficient (*r _b_*) relative to the liver-red module eigengene, and the cross-platform sample correlation coefficients are shown in the following table.

| Illumina ProbeID | Symbol | Liver Module | *c* | *F_s_*  p-value | *r _b_ (liver- red)* | Affy ProbeSetID | *r*(cross-platform) |
| --- | --- | --- | --- | --- | --- | --- | --- |
| 460112 | *Acadm* | Red | 0.59 | 0.004 | -0.89 | 10502951 | 0.56 |
| 540020 | *Crat* | Red | 0.84 | 2E-04 | -0.66 | 10481474 | 0.60 |
| 2370110 | *Cyp4a14* | Red | 0.96 | 1E-06 | -0.83 | 10515187 | 0.85 |
| 6520735 | *Tnfrsf1a* | Red | 0.83 | 5E-05 | 0.95 | 10541895 | 0.74 |

**Supplemental Figure S5: Transcript abundance profiles for circadian rhythm genes**

The transcript abundance profiles for 12 genes associated with circadian rhythm in adipose (A), heart (B), kidney (C), liver on the Illumina platform (D), and liver on the Affymetrix platform (E) are shown. The graphical features of the eigengene plots of Figure 4 apply to these gene plots. Profiles are coloured by module membership. For each gene, the probe identifiers, module assignment, intraclass correlation coefficient (*c*), *F_s_* p-value, between-mouse Pearson correlation coefficients (*r _b_*) relative to the adipose-red (a-red), heart-blue (h-blue), kidney-brown (k-brown), liver-blue (l-blue), and liver-black (l-black) module eigengenes, and the cross-platform sample correlation coefficients for liver are shown in the table below. To make the reading of the correlation coefficients easier, they are in bold print for the eigengene of the module to which the gene belongs and they are in italics for eigengenes of the same tissue as the gene profile where the gene does not belong to the module. Otherwise, the correlations are printed in grey.

| Illumina ProbeID | Tissue | Symbol | Adipose mod | Heart Module | Kidney Mod | Liver Mod | *c* | *F_s_* p-value | *r _b_(a-red)* | *r _b_(h-blue)* | *r _b_(k-brown)* | *r _b_(l-blue)* | *r _b_(l-black)* | Affy ProbeID | *r (cross-platform)* |
| --- | --- | --- | --- | --- | --- | --- | --- | --- | --- | --- | --- | --- | --- | --- | --- |
| 2510142 | Adipose | *Gadd45g* | red | blue | brown | black | 0.53 | 0.02 | **0.92** | 0.73 | 0.86 | 0.82 | 0.77 |  |  |
|  | Heart |  |  |  |  |  | 0.63 | 3.E-3 | 0.83 | **0.89** | 0.66 | 0.71 | 0.55 |  |  |
|  | Kidney |  |  |  |  |  | 0.97 | 6.E-6 | 0.91 | 0.71 | **0.92** | 0.71 | 0.91 |  |  |
|  | Liver |  |  |  |  |  | 1.00 | 0 | 0.88 | 0.59 | 0.83 | *0.65* | **0.95** | 10405211 | **0.96** |
| 4850164 | Adipose | *Mt1* | red | blue | pink | black | 0.33 | 0.08 | **0.84** | 0.72 | 0.75 | 0.59 | 0.77 |  |  |
|  | Heart |  |  |  |  |  | 0.59 | 0.01 | 0.68 | **0.87** | 0.45 | 0.47 | 0.56 |  |  |
|  | Kidney |  |  |  |  |  | 0.89 | 1.E-4 | 0.48 | 0.14 | *0.54* | 0.25 | 0.77 |  |  |
|  | Liver |  |  |  |  |  | 1.00 | 0 | 0.86 | 0.68 | 0.84 | *0.56* | **0.91** | 10574027 | **0.91** |
| 3360138 | Adipose | *Pim3* | grey | blue | brown | black | 0.2 | 0.20 | *0.67* | 0.48 | 0.64 | 0.43 | 0.69 |  |  |
|  | Heart |  |  |  |  |  | 0.32 | 0.04 | 0.79 | **0.94** | 0.63 | 0.71 | 0.54 |  |  |
|  | Kidney |  |  |  |  |  | 0.86 | 6.E-5 | 0.70 | 0.74 | **0.63** | 0.52 | 0.61 |  |  |
|  | Liver |  |  |  |  |  | 0.98 | 0 | 0.91 | 0.64 | 0.90 | *0.73* | **0.92** | 10426110 | **0.91** |
| 6400390 | Adipose | *Map3k6* | red | blue | brown | black | 0.42 | 0.04 | **0.90** | 0.63 | 0.91 | 0.89 | 0.73 |  |  |
|  | Heart |  |  |  |  |  | 0.54 | 5.E-3 | 0.82 | **0.95** | 0.66 | 0.79 | 0.58 |  |  |
|  | Kidney |  |  |  |  |  | 0.83 | 9.E-5 | 0.90 | 0.54 | **0.92** | 0.64 | 0.91 |  |  |
|  | Liver |  |  |  |  |  | 0.54 | 3.E-3 | 0.84 | 0.58 | 0.88 | *0.59* | **0.86** | 10508829 | **0.57** |
| 2190048 | Adipose | *Fkbp5* | red | blue | brown | blue | 0.77 | 6.E-4 | **0.92** | 0.64 | 0.94 | 0.88 | 0.84 |  |  |
|  | Heart |  |  |  |  |  | 0.60 | 2.E-3 | 0.62 | **0.87** | 0.45 | 0.66 | 0.43 |  |  |
|  | Kidney |  |  |  |  |  | 0.82 | 7.E-5 | 0.85 | 0.69 | **0.87** | 0.91 | 0.76 |  |  |
|  | Liver |  |  |  |  |  | 0.96 | 2.E-6 | 0.88 | 0.75 | 0.85 | **0.95** | *0.66* | 10449452 | **0.95** |
| 3290315 | Adipose | *Per1* | red | blue | brown | blue | 0.53 | 0.02 | **0.82** | 0.76 | 0.78 | 0.72 | 0.62 |  |  |
|  | Heart |  |  |  |  |  | 0.84 | 3.E-4 | 0.66 | **0.72** | 0.56 | 0.55 | 0.39 |  |  |
|  | Kidney |  |  |  |  |  | 0.95 | 1.E-5 | 0.67 | 0.60 | **0.59** | 0.39 | 0.68 |  |  |
|  | Liver |  |  |  |  |  | 0.78 | 4.E-4 | 0.81 | 0.82 | 0.73 | **0.81** | *0.47* | 10377439 | **0.85** |
| 3450273 | Adipose | *Errfi1* | red | blue | grey | black | 0.81 | 2.E-4 | **0.64** | 0.57 | 0.58 | 0.33 | 0.66 |  |  |
|  | Heart |  |  |  |  |  | 0.52 | 0.01 | 0.75 | **0.79** | 0.60 | 0.53 | 0.56 |  |  |
|  | Kidney |  |  |  |  |  | 0.50 | 4.E-3 | 0.51 | 0.63 | *0.41* | 0.33 | 0.49 |  |  |
|  | Liver |  |  |  |  |  | 0.95 | 4.E-5 | 0.78 | 0.71 | 0.67 | *0.47* | **0.71** | 10510574 | **0.77** |
| 1400131 | Adipose | *Sgk1* | red | blue | brown | grey | 0.67 | 3.E-3 | **0.76** | 0.81 | 0.66 | 0.61 | 0.61 |  |  |
|  | Heart |  |  |  |  |  | 0.6 | 4.E-3 | 0.49 | **0.60** | 0.37 | 0.30 | 0.27 |  |  |
|  | Kidney |  |  |  |  |  | 0.91 | 2.E-5 | 0.59 | 0.55 | **0.50** | 0.35 | 0.59 |  |  |
|  | Liver |  |  |  |  |  | 0.90 | 8.E-5 | 0.28 | 0.34 | 0.22 | *0.11* | *0.15* | 10362073 | **0.83** |
| 6400706 | Adipose | *Cdkn1a* | gold | blue | brown | blue | 0.40 | 0.05 | *0.62* | 0.33 | 0.65 | 0.62 | 0.56 |  |  |
|  | Heart |  |  |  |  |  | 0.70 | 9.E-4 | 0.66 | **0.88** | 0.44 | 0.56 | 0.39 |  |  |
|  | Kidney |  |  |  |  |  | 0.92 | 1.E-5 | 0.85 | 0.58 | **0.80** | 0.65 | 0.86 |  |  |
|  | Liver |  |  |  |  |  | 0.99 | 0.00 | 0.86 | 0.76 | 0.85 | **0.89** | 0.74 | 10443463 | **0.95** |
| 2480195 | Adipose | *Angptl4* | magenta | pink | brown | blue | 0.00 | 0.56 | *0.29* | -0.12 | 0.32 | 0.04 | 0.39 |  |  |
| 6760593 | Heart |  |  |  |  |  | 0.00 | 0.14 | 0.42 | *0.56* | 0.39 | 0.47 | 0.34 |  |  |
| 6760593 | Kidney |  |  |  |  |  | 0.89 | 3.E-5 | 0.95 | 0.77 | **0.93** | 0.83 | 0.81 |  |  |
| 2480195 | Liver |  |  |  |  |  | 0.85 | 1.E-4 | 0.81 | 0.66 | 0.82 | **0.83** | *0.62* | 10450038 | **0.82** |
| 6860121 | Adipose | *Dusp1* | red | grey | brown | black | 0.27 | 0.12 | **0.64** | 0.39 | 0.57 | 0.32 | 0.67 |  |  |
|  | Heart |  |  |  |  |  | 0.44 | 3.E-2 | 0.57 | *0.54* | 0.60 | 0.63 | 0.50 |  |  |
|  | Kidney |  |  |  |  |  | 0.97 | 1.E-5 | 0.73 | 0.58 | **0.70** | 0.49 | 0.74 |  |  |
|  | Liver |  |  |  |  |  | 0.97 | 0 | 0.50 | 0.18 | 0.59 | *0.38* | **0.61** | 10449284 | **0.97** |
| 3390593 | Adipose |  | red | turquoise | gold | pink | 0.41 | 0.05 | **0.88** | 0.79 | 0.77 | 0.77 | 0.63 |  |  |
|  | Heart | *Per2* |  |  |  |  | 0.66 | 3.E-3 | 0.63 | *0.85* | 0.49 | 0.57 | 0.47 |  |  |
|  | Kidney |  |  |  |  |  | 0.62 | 5.E-3 | -0.27 | -0.22 | *-0.21* | -0.42 | -0.22 |  |  |
|  | Liver |  |  |  |  |  | 0.88 | 1.E-4 | -0.45 | -0.15 | -0.50 | *0.35* | *0.61* | 10356601 | **0.80** |

**Supplemental Figure S6: Transcript abundance profiles for growth-hormone regulated genes in kidney and liver**

The liver transcript abundance profiles for growth-hormone responsive genes identified in Chen et al. (2009) are shown. From top to bottom, the profiles are for *Socs2*, *Gadd45g*, *Cish*, and *Bcl6*. The graphical features of the eigengene plots of Figure 4 apply to these gene plots. Profiles are coloured by module membership. For each gene, the probe identifiers, module assignment, intraclass correlation coefficient (*c*), *F_s_* p-value, between-mouse Pearson correlation coefficients (*r _b_*) relative to the liver-black, liver-magenta, kidney-brown, and kidney-pink module eigengenes, and the cross-platform sample correlation coefficient for liver are shown in the following table.

| ProbeID | Symbol | Kidney Module | Liver Module | *c* | *F_s_*  p-value | *r _b_ (liver-black)* | *r _b_ (liver-magenta)* | *r _b_ (kidney-brown)* | *r _b_ (kidney-pink)* | *Affy ProbeID* | *r (cross-platform)* |
| --- | --- | --- | --- | --- | --- | --- | --- | --- | --- | --- | --- |
| 4760692 | *Socs2* | pink | magenta | 0.98 | 0 | 0.63 | -0.67 | 0.19 | 0.70 | 10372069 | 0.71 |
| 2510142 | *Gadd45g* | brown | black | 1.00 | 0 | 0.84 | -0.53 | 0.83 | 0.68 | 10405211 | 0.96 |
| 840315 | *Cish* | pink | magenta | 1.00 | 0 | 0.71 | -0.95 | 0.31 | 0.96 | 10588577 | 0.81 |
| 940100 | *Bcl6* | pink | magenta | 0.99 | 0 | 0.47 | 0.68 | -0.08 | -0.69 | 10438738 | 0.76 |

**Supplemental Figure S7: Transcript abundance profiles for variable genes in adipose tissue**

The within-mouse variation patterns for the transcripts *Sfrp5*, *Trp53inp2*, *Lep*, *Mest*, and *Bmp3* (from top to bottom) are correlated, suggesting they share a similar heterogeneous spatial distribution within the inguinal fat pad (A). The graphical features of the eigengene plots of Figure 4 apply to these gene plots. Even though the significance of between-mouse *F_s_* statistics for these genes is marginal, their between-mouse patterns are correlated and also correlate with body weight (B). For each gene, the probe identifier, module assignment, intraclass correlation coefficient (*c*), *F_s_* p-value, and the between-mouse Pearson correlation coefficients (*r _b_*) relative to body weight and the adipose-magenta module eigengene are shown in the following table.

| ProbeID | Symbol | Adipose Module | *c* | *F_s_*  p-value | *r _b_*  *(body-weight)* | *r _b_ (adipose-magenta)* | *r _w_ (adipose-magenta)* |
| --- | --- | --- | --- | --- | --- | --- | --- |
| 2810114 | *Sfrp5* | magenta | 0.06 | 0.27 | 0.45 | -0.92 | -0.85 |
| 2690435 | *Trp53inp2* | magenta | 0.43 | 0.03 | 0.41 | -0.89 | -0.93 |
| 4010053 | *Lep* | magenta | 0.00 | 0.41 | 0.50 | -0.78 | -0.82 |
| 6620292 | *Mest* | magenta | 0.02 | 0.29 | 0.47 | -0.62 | -0.83 |
| 4230673 | *Bmp3* | magenta | 0.00 | 0.85 | 0.25 | -0.70 | -0.88 |

**Supplemental Figure S8: Transcript abundance profiles for variable brown fat signature genes in white fat tissue**

The transcript abundance profiles for *Ucp1* (A), *Cidea* (B), *Prdm16* (C), and *Ckm* (D) are shown. The graphical features of the eigengene plots of Figure 4 apply to these gene plots. For each gene, the probe identifier, module assignment, intraclass correlation coefficient (*c*), *F_s_* p-value, and the between-mouse Pearson correlation coefficients (*r _b_*) relative to the adipose-gold and adipose-brown module eigengenes are shown in the following table.

| ProbeID | Symbol | Adipose Module | *c* | *F_s_*  p-value | *r _b_ (adipose-gold)* | *r _b_ (adipose-brown)* | *r _w_ (adipose-gold)* | *r _w_ (adipose-brown)* |
| --- | --- | --- | --- | --- | --- | --- | --- | --- |
| 6020368 | *Ucp1* | gold | 0.00 | 0.85 | 0.70 | -0.53 | 0.36 | -0.54 |
| 4560020 | *Cidea* | gold | 0.00 | 0.91 | 0.93 | -0.27 | 0.55 | -0.46 |
| 101190239 | *Prdm16* | grey | 0.00 | 0.56 | 0.86 | -0.18 | 0.74 | -0.00 |
| 1450524 | *Ckm* | brown | 0.42 | 0.01 | -0.22 | 0.95 | -0.23 | 0.91 |

**Supplemental Figure S9: Transcript abundance profiles showing region-specific variation of gene expression in heart**

The transcript abundance profiles for *Myl4*, *Myl7* (top to bottom of left panel of (A)), *Tbx5*, *Gja5*, and *Bmp10* (top to bottom of right panel of (A)), and *Gata4* (B) are shown. The graphical features of the eigengene plots of Figure 4 apply to these gene plots. For each gene, the probe identifier, module assignment, intraclass correlation coefficient (*c*), *F_s_* p-value, and the between-mouse Pearson correlation coefficients (*r _b_*) relative to the heart-green and heart-red module eigengenes are shown in the following table.

| ProbeID | Symbol | Heart Module | *c* | *F_s_*  p-value | *r _b_ (heart-green)* | *r _b_ (heart- red)* | *r _w_ (heart-green)* | *r _w_ (heart-red)* |
| --- | --- | --- | --- | --- | --- | --- | --- | --- |
| 6860288 | *Myl4* | green | 0.00 | 0.36 | 0.63 | 0.29 | 0.93 | 0.8 |
| 2480541 | *Myl7* | green | 0.00 | 0.29 | 0.64 | 0.3 | 0.95 | 0.82 |
| 106840364 | *Tbx5* | green | 0.00 | 0.82 | 0.85 | 0.16 | 0.98 | 0.82 |
| 1740026 | *Gja5* | green | 0.00 | 0.95 | 0.72 | -0.13 | 0.99 | 0.85 |
| 3060068 | *Bmp10* | green | 0.00 | 0.89 | 0.39 | -0.43 | 0.81 | 0.54 |
| 2690609 | *Gata4* | red | 0.02 | 0.36 | -0.49 | -0.76 | -0.84 | -0.86 |

**Supplemental Figure S10: Transcript abundance profiles for androgen-regulated genes variable in the kidney**

The transcript abundance profiles in kidney for several genes reported to be androgen-responsive are shown (from top to bottom: *Hsd11b1*, *Hsd17b11*, *Srd5a2*, *Gusb*, and *Prlr*). *Gusb* is assigned the colour grey since it was not among the 2500 most variable genes in kidney. *Prlr* is negatively correlated with the other transcripts. The graphical features of the eigengene plots of Figure 4 apply to these gene plots. For each gene, the probe identifier, module assignment, intraclass correlation coefficient (*c*), *F_s_* p-value, and the between-mouse Pearson correlation coefficient (*r _b_*) relative to the kidney-green module eigengene are shown in the following table.

| ProbeID | Symbol | Kidney Module | *c* | *F_s_*  p-value | *r _b_ (kidney-green)* |
| --- | --- | --- | --- | --- | --- |
| 5550408 | *Hsd11b1* | green | 0.79 | 1E-04 | 0.88 |
| 4540239 | *Hsd17b11* | green | 0.89 | 5E-05 | 0.95 |
| 6840433 | *Srd5a2* | green | 0.86 | 1E-04 | 0.91 |
| 7160053 | *Gusb* | grey | 0.50 | 0.02 | 0.73 |
| 1990097 | *Prlr* | green | 0.98 | 1E-06 | -0.58 |

**Supplemental Figure S11: Transcript abundance profile for *Cfd* gene in kidney**

The transcript abundance profile in kidney for *Cfd* is shown. The graphical features of the eigengene plots of Figure 4 apply to this gene plot. The probe identifier, module assignment, intraclass correlation coefficient (*c*), *F_s_* p-value, and the between-mouse Pearson correlation coefficient (*r _b_*) relative to the kidney-black module eigengene are shown in the following table.

| ProbeID | Symbol | Kidney Module | *c* | *F_s_*  p-value | *r _b_ (kidney-black)* | *r _w_ (kidney-black)* |
| --- | --- | --- | --- | --- | --- | --- |
| 2320736 | *Cfd* | black | 0.11 | 0.001 | 0.91 | 0.93 |

# References

**1. Cui X, Hwang J, Qiu J, Blades N, Churchill G: Improved statistical tests for differential gene expression by shrinking variance components estimates. *Biostatistics* 2005, 6(1):59-75.**

**2. Lu X, Perkins DL: Re-sampling strategy to improve the estimation of number of null hypotheses in FDR control under strong correlation structures. *BMC Bioinformatics* 2007, 8:157.**

**3. Chen Y, Lin G, Huo JS, Barney D, Wang Z, Livshiz T, States DJ, Qin ZS, Schwartz J: Computational and functional analysis of growth hormone (GH)-regulated genes identifies the transcriptional repressor B-cell lymphoma 6 (Bc16) as a participant in GH-regulated transcription. *Endocrinology* 2009, 150(8):3645-3654.**
