# Supplementary material for: Stochastic variation of transcript abundance in C57BL/6J mice
Source: BMC Genomics. 2011 Mar 30;12:167. doi: 10.1186/1471-2164-12-167 (PMC3082245; doi:10.1186/1471-2164-12-167)
Supplement: Additional file 10 — Supplemental Table S3. Within-mouse correlation statistics for selected genes in adipose. [file 1471-2164-12-167-S10.DOC]

| ProbeID | Symbol | Adipose Module | *c* | *F_s_*  p-value | *r _b_ (adipose-green)* | *r _b_ (adipose-magenta)* | *r _w_ (adipose-green)* | *r _w_ (adipose-magenta)* |
| --- | --- | --- | --- | --- | --- | --- | --- | --- |
| 6660390 | *Ccl6* | green | 0.00 | 0.62 | 0.96 | 0.77 | 0.88 | -0.91 |
| 4610725 | *Ccl9* | green | 0.17 | 0.14 | 0.91 | 0.73 | 0.94 | -0.80 |
| 2570035 | *Cd8b1* | magenta | 0.00 | 1.00 | 0.37 | 0.81 | -0.69 | 0.99 |
| 4780450 | *Elf1* | magenta | 0.00 | 0.97 | 0.67 | 0.94 | -0.58 | 0.99 |
| 4010053 | *Lep* | magenta | 0.00 | 0.41 | -0.53 | -0.78 | 0.73 | -0.82 |
| 2690435 | *Trp53inp2* | magenta | 0.43 | 0.03 | -0.72 | -0.89 | 0.57 | -0.93 |

**Supplemental Table S3: *Within-tissue correlation of eigengene transcript abundance profiles.*** For each gene of Figure 5, this table shows module assignment, intraclass correlation coefficient (*c*), *F_s_* permutation p-value, and between- (*r _b_*) and within-mouse (*r _w_*) Pearson correlation coefficients relative to the adipose-green and adipose-magenta module eigengenes.
