# Supplementary material for: Stochastic variation of transcript abundance in C57BL/6J mice
Source: BMC Genomics. 2011 Mar 30;12:167. doi: 10.1186/1471-2164-12-167 (PMC3082245; doi:10.1186/1471-2164-12-167)
Supplement: Additional file 15 — Supplemental Table S4. Transcript abundance variation statistics for Pritchard et al (2001) dataset. [file 1471-2164-12-167-S15.DOC]

#

|  |  | **Kidney** | **Liver** | **Testes** |
| --- | --- | --- | --- | --- |
| **(A)** | **Total Genes** | 5269 | 5231 | 5198 |
| **(B)** | **Variable Genes** |  |  |  |
|  | ***α*=0.05** | 2592(49%) | 2551(49%) | 2687(52%) |
|  | ***α*=0.01** | 2562(49%) | 2513(48%) 2636(51%) | 2636(51%) |
|  | *α*=1e‑4 | 2554(48%) | 2499(48%) | 2619(50%) |
| **(C)** | **Between-Mouse Variation** | |  |  |
|  | **FWER (p < 0.05 )** | 44(1%) | 9(0%) | 77(2%) |
|  | **FDR (p < 0.10 )** | 715(14%) | 38(1%) | 1794(35%) |
|  | **1 – π_0_** | 53% | 63% | 77% |
| **(D)** | **Maximal Fold Change** | |  |  |
|  | **>1.5** | 2422(46%) | 2712(52%) | 5109(98%) |
|  | **>2.0** | 966(18%) | 401(8%) | 3934(76%) |
|  | **>3.0** | 220(4%) | 31(1%) | 1016(20%) |

**Supplementary Table S4: *Variability of transcript abundance for Pritchard et al. 2001 experiment***. This table contains summary statistics for the Pritchard et al. (2001) experiment. The numbers of probes on the array (A) and the numbers of variable genes based on the 0.05, 0.01 and 0.0001 tails of the scaled *χ_2_*(47) distribution are shown (B). The numbers of genes with significant between-mouse variation, based on an *F_s_* test with a family-wise adjusted error rate (p<0.05; Sidak step-down method, Westfall and Young ) and a false discovery rate (p<0.10; Benjamini and Hochberg ) are provided. The estimated proportions of differentially expressed genes, 1 – π_0_, using the q-value method (Storey and Tibshirani,) are also provided (C). The numbers of genes with large maximal fold-change between mice are shown (D). Percentages in (A), (C), and (D) based on total number of probes on the array are given in parentheses.

# References

**1. Pritchard C, Hsu L, Delrow J, Nelson P: Project normal: Defining normal variance in mouse gene expression. *Proceedings of the National Academy of Sciences, USA* 2001, 98:13266-13271.**

**2. Cui X, Churchill G: How many mice and how many arrays? Replication in mouse cDNA microarray experiments. In: *Methods of Microarray Data Analysis III, Papers from CAMDA ’02.* Edited by SM JKaL; 2003.**

**3. Westfall PH, Young SS: Resampling-based Multiple Testing. New York.: Wiley; 1993.**

**4. Benjamini Y, Hochberg Y: Controlling the false discovery rate: a practical and powerful approach to multiple testing. *J Roy Statist Soc Ser B (Methodological)* 1995, 57:289-300.**

**5. Storey J, Tibshirani R: Statistical significance for genomewide studies. *Proceedings of the National Academy of Sciences, USA* 2003, 100(16):9440-9445.**
